# Photonic scaffolds as ultrahigh-openness on-chip hollow-core waveguides for quantum photonics and optofluidics

**DOI:** 10.1038/s41467-026-75873-1

**Published:** 2026-07-28

**Authors:** Wenqin Huang, Diana Pereira, Matthias Zeisberger, Hala Said, Esteban Gómez-López, Jun Sun, Oliver Benson, Markus A. Schmidt

**Affiliations:** 1 Fiber Photonics, Leibniz Institute of Photonic Technology, Albert-Einstein-Str. 9, Jena, Germany; 2 Abbe Center of Photonics and Faculty of Physics, Friedrich-Schiller-University Jena, Max-Wien-Platz 1, Jena, Germany; 3 i3N & Physics Department, University of Aveiro, Campus de Santiago, Aveiro, Portugal; 4 Department of Physics, Humboldt-Universität zu Berlin, Newtonstraße 15, Berlin, Germany; 5 Otto Schott Institute of Materials Research (OSIM), Friedrich Schiller University Jena, Lessingstrasse 12, Jena, Germany

**Keywords:** Optical materials and structures, Integrated optics

## Abstract

On-chip hollow-core waveguides enable strong light-matter interaction in gases and liquids with high integration density, yet limited side access to the core restricts their use in diffusion-driven processes. We introduce a distinct class of on-chip hollow-core waveguides that confines light in a scaffold membrane geometry composed predominantly of air, reaching cladding-openness fraction up to 80% while maintaining optical losses comparable to fully enclosed waveguides. Light guidance in the photonic scaffold is enabled by the combination of anti-resonant confinement with Bloch-mode formation in a segmented structure. High-precision 3D nanoprinting, extensive optical characterization and a resonator-based theoretical model confirm the guiding principle and loss behavior. We demonstrate application relevance through enhanced molecular diffusion, highly integrated optofluidic spectroscopy, and efficient single-photon transmission enabled by the open scaffold geometry and air-dominant guidance.

## Abstract (web summary)

On-chip hollow-core waveguides could advance quantum optics and optofluidics, but limited core access hampers light-matter interactions. Here, the authors demonstrate 3D-printed photonic scaffold waveguides enabling low-loss guidance, fast diffusion, spectroscopy, and single-photon transmission.

## Introduction

Integrated photonic platforms are essential in key areas of modern society, such as quantum technologies, artificial intelligence, and analytical chemistry, where miniaturization reduces device size, enhances interaction, minimizes sample consumption, and boosts sensitivity.

Here, on-chip waveguides offer a compelling platform with extended light-matter interaction lengths and high integration density, enabling essential quantum-related applications such as the single photon nonlinearities^1^ or entangled photon pair generation^2^, and the detection of ultra-low analyte concentrations^3–5^, including individual diffusing species^6^. The mentioned applications pose challenges for waveguide design, as the potential core medium often has a lower refractive index than the cladding, preventing light confinement via total internal reflection.

Light confinement in low-index cores is a key focus in modern fiber optics, leading to the development of hollow-core fibers (HCFs) that guide light through mechanisms such as photonic bandgaps^7^ or the antiresonant effect^8^. The latter designs offer ultra-low loss, broad bandwidth, and low dispersion with minimal structural complexity^9^, and are now being commercialized for ultralow-latency inter-data-center links^10^. Moreover, their strong light-matter interaction within the central core makes HCFs highly attractive for applications in quantum science^11^, chemical analysis^12^, and nanoanalytics^13^.

In contrast, concepts for on-chip hollow-core waveguides (HCWs) have remained limited for a long time to anti-resonant reflecting optical waveguides (ARROWs), used for spectroscopic^14^ and analytical applications^15^. Recent advances in 3D nanoprinting enable the transfer of fiber-related concepts to planar photonics, boosting interest in on-chip HCWs. Beyond the light cage geometry^16–18^, antiresonant membrane HCWs (mHCWs)^19^ have emerged as a promising solution, with applications in spectroscopy detection^20^, higher-order mode excitation^21^, and nanoparticle tracking analysis^22^.

While effective in many applications, HCWs face serious limitations in scenarios requiring side access to the core. A representative example is vapor-based quantum technologies, where filling centimeter-long HCFs with low-pressure alkali vapor relies on diffusion and takes several months to reach adequate core concentrations^23,24^. Attempts to accelerate this process by drilling holes into the cladding^25,26^ provide only modest improvements while increasing modal losses. Membrane-HCWs offer a highly promising on-chip platform in this context due to excellent light guiding properties^19^, although they still suffer from restricted lateral accessibility, limiting their suitability for applications involving dynamic or diffusing matter. This underscores the need for next-generation on-chip HCW solutions offering maximal lateral accessibility for diffusion-driven applications and rapid core material exchange.

In this work, we present an unexplored class of membrane-based on-chip HCW -*the Photonic Scaffold*- that confines light in air-dominated scaffold geometries with an ultrahigh cladding-openness fraction of up to 80%, enabling direct lateral access to the core. Using high-precision 3D nanoprinting, we realize and characterize two distinct implementations of the photonic scaffold HCW and perform comprehensive structural and optical analysis through experiments and simulations, supported by a resonator-based theoretical model. To demonstrate application relevance, we validate the platform’s suitability for integrated optofluidics and quantum photonics through dye diffusion experiments, absorption spectroscopy, and single-photon transmission.

## Results

### Concept

**Fig. 1:**
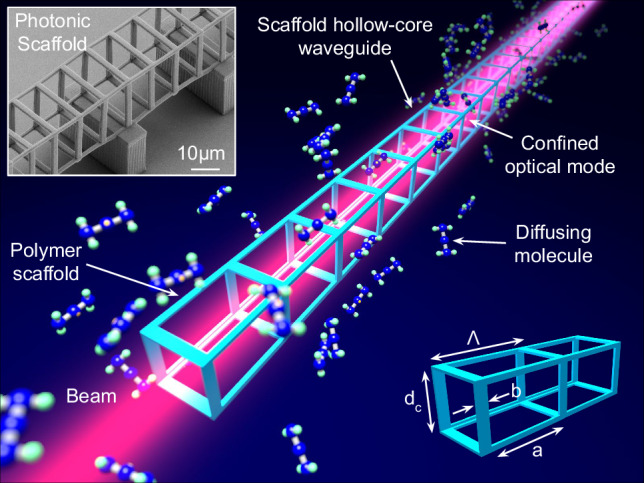
Artistic illustration of the segmented on-chip hollow-core waveguide - *the Photonic Scaffold* - that includes ultrahigh cladding-openness fraction, allowing light guidance in an air-dominated geometry. The displayed molecules are schematic representations of Rhodamine 6G (R6G), used in diffusion-related experiments (the color coding highlights the different functional parts of the molecule). The top left inset shows a scanning electron micrograph (SEM) of a 3D nanoprinted waveguide with a cladding-openness fraction of 80%. The bottom right image shows the key geometric parameters of waveguide geometry.

The membrane section of the scaffold HCW (sHCW) section generally consists of a thin, square polymer membrane of thickness *t* surrounding a central hollow core (Fig. 1). Light confinement is governed by the anti-resonant effect, where interference within the membrane enhances interface reflectivity, enabling low-loss propagation of the core mode^8^. In a ray-optic picture, the large core diameter (*d*_*c*_ > 10 μ*m*) leads to shallow incidence angles at the core-membrane interface, further increasing reflectivity and reducing modal losses. Coupling between the core mode and the high-index modes of the dielectric membrane results in a distinct spectral signature—alternating bands of high and low transmission. Resonances, observed as transmission dips, occur at wavelengths where the dispersions of the core and membrane modes intersect. These resonance wavelengths can be approximated using the cutoff condition for the mode of a symmetric high-index slab waveguide:

1 $${\lambda }_{{{{\rm{res}}}}}=\frac{2t}{m}\sqrt{{n}_{m}^{2}-{n}_{c}^{2}}$$

where *n*_*m*_ and *n*_*c*_ are the refractive indices of the membrane and core, respectively, and *m* is the mode order.

As mentioned, the large core extension results in shallow reflection angles, enabling the formation of modes with minimal diffraction over tens of micrometers. This characteristic suggests that periodically omitting defined sections of the membrane along the waveguide axis does not significantly increase modal losses *γ*, leading to the photonic scaffold design. Such selective removal facilitates lateral access to the core and is characterized by the openness fraction of the membraned cladding, defined as the ratio of the opening area *a* to the total membrane area within one period *Λ* (Fig. 1):

2 $$f=\frac{{A}_{{{{\rm{opening}}}}}}{{A}_{{{{\rm{total}}}}}}=\frac{a{d}_{c}}{\Lambda {d}_{c}}=\frac{a}{\Lambda }$$

To systematically explore the impact of introducing openings into the membranes of the sHCW, two types of Photonic Scaffold geometries with different longitudinal distributions of the openings are defined and investigated experimentally:

*Type I Scaffold*: A periodic sequence of openings with a symmetric membrane arrangement that fully encloses the central hollow core.

*Type II Scaffold*: A periodic sequence of openings featuring a half-period shift between vertical and horizontal openings.

### Physics of mode formation

To understand mode formation in the Photonic Scaffold, a segmented leaky-mode slab-waveguide resonator model is developed that accounts for both axial periodicity and transverse power dissipation. This approach treats the Photonic Scaffold as a periodically modulated leaky HCW, where the structural periodicity leads to the formation of dissipative Bloch modes and an associated band structure that includes stop bands^27^. By applying longitudinal boundary conditions, the model enables simulating the dissipative band edge eigenstates, which govern the overall modal behavior. Note that the slab waveguide model accurately describes the loss of a square-shaped HCW design, as *γ*_HCW_ ≈ 2*γ*_SW_ (*γ*_SW_: slab waveguide loss, see Supplementary Note 2 for further details)^19^.

According to ref. ^28^, the bandwidth of a stop band in the limit of small refractive index difference Δ*n* is given by

3 $$\Delta \lambda \approx \frac{2}{\pi }\,\Delta n$$

As the large core size results in an effective mode index close to that of air (${{{\rm{Re}}}}({n}_{{{{\rm{eff}}}}})\approx 0.9999$, $\Delta n=(1-{{{\rm{Re}}}}({n}_{{{{\rm{eff}}}}}))=1{0}^{-4}$, see Supplementary Note 2), extremely narrowband stop bands of

4 $$\delta \lambda \approx 1{0}^{-4}$$

are obtained, allowing to solely focus on a single band-edge mode to capture the modal properties. Moreover, as described in ref. ^28^, the total reflectivity of a band-edge mode can be approximated as

5 $${R}_{N}\approx \frac{\Delta {n}^{2}}{\Delta {n}^{2}+{(2/N)}^{2}}$$

with the number of unit cells *N*. For Δ*n* ≈ 10^−4^, the total reflectivity remains below 4% even for *N* = 2000, which exceeds experimental conditions and allows periodicity-induced reflection to be safely neglected.

The geometry is defined by the membrane thickness *t*, the unit cell extension (i.e., period *Λ*) and filling fraction *f*, with the simulation domain terminated longitudinally by perfect magnetic or electric conductors, resulting in TE- or TM-polarized modes. As detailed in Supplementary Note 2, the losses of the segmented slab waveguide arise from power dissipation of the band-edge eigenstates and are directly linked to the Q-factor of the resonances via ${{{\rm{Im}}}}({n}_{{{{\rm{eff}}}}})\approx 1/(2Q)$, leading to:

6 $${\gamma }_{{{{\rm{SW}}}}}\,[{{{\rm{dB/mm}}}}]=\frac{27.3}{Q\cdot \lambda \,[{{{\rm{mm}}}}]}$$

**Fig. 2:**
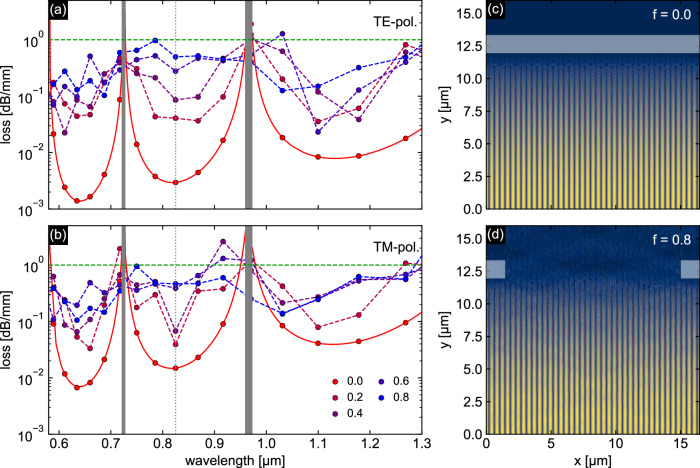
Cladding-openness fraction analysis using the resonator model. The left column shows the spectral distribution of simulated dissipative band-edge eigenstates for various cladding-openness fractions ((**a**) TE polarization, (**b**) TM polarization; simulation parameters are provided in Supplementary Table 1). Different colors indicate different cladding-openness fractions, as shown in the legend. To improve visibility, the points of the discrete eigenstates are connected by dashed lines. The solid red line represents results from simulations of the dispersion of the fundamental leaky mode of the square-shaped membrane HCW, referring to the case of *f* = 0. The horizontal green dashed lines mark a loss level of 1 dB/mm. Gray rectangles mask the resonance regions, corresponding to high-loss domains caused by modal anticrossing. The right column shows the absolute electric field magnitude of a selected eigenstate at *λ*_0_ = 825 nm (indicated by vertical dotted gray lines in (**a**, **b**)) for two cladding-openness fractions in TE polarization ((**c**) *f* = 0, (**d**) *f* = 0.8). The semitransparent white areas indicate the polymeric material. The color scale ranges linearly from the maximum value (yellow) to zero (dark blue).

This model enables estimation of the loss in the periodic environment as a function of geometric parameters for both TE and TM polarizations (see Fig. 2, simulation parameters are listed in Supplementary Table 1). To validate the approach, the loss of a gapless configuration (red dots, *f* = 0) was compared with full mode simulation results (red solid curve), showing excellent agreement for both polarizations. As the cladding-openness fraction (i.e., gap extend) increases, the dissipative band-edge eigenstates show rising loss values, yet losses remain below 1 dB/mm in nearly all situations. This is significant from the experimental perspective, as typical measured losses of mHCWs are of the order of 1 dB/mm and are attributed mainly to surface roughness rather than insufficient confinement. Even for *f* = 0.8 (blue dots in Fig. 2a, b), the loss remains at or below 1 dB/mm (horizontal green dashed lines in Fig. 2a, b), despite the structure being mostly air.

This behavior is also evident in the electric field distribution of the band-edge modes at a representative wavelength (Fig. 2c, d): even with the large gap size at *f* = 0.8, the field is still strongly confined to the core region (Fig. 2d), resulting in small power dissipation, i.e., low modal loss, and indicating the performance potential of the Photonic Scaffold.

Note that the non-monotonic spectral variation of the loss (dots in Fig. 2a, b) likely originates from gap-induced longitudinal resonances, as illustrated by the complex field distribution outside the core region in Fig. 2d. Consistent with the discussion above, a very small polarization-dependent spectral difference between the corresponding band-edge eigenstates is observed (e.g., TE: 824.883 nm, TM: 824.896 nm), confirming that only extremely narrow and practically negligible stop gaps are formed. It is also important to note that the discrete nature of the eigenstates results from the use of periodic boundary conditions at the edges of a single unit cell. When a larger simulation domain containing multiple unit cells is used, the density of resonances increases, forming a quasi-continuous transmission band with individual resonances that are no longer experimentally resolvable.

### Selected examples of implemented sHCWs

To experimentally validate the feasibility of high-cladding-openness fraction sHCWs, we utilized a commercial 3D nanoprinter (GT2, Nanoscribe) to fabricate both Type I and Type II Photonic Scaffolds (see Sec. 4 for fabrication details). This implementation approach allowed to systematically vary structural parameters—such as the opening length—unlike conventional techniques. The sHCWs were horizontally nanoprinted onto a silicon chip and elevated using solid vertical supports, ensuring structural stability and eliminating chip-induced effects.

**Fig. 3:**
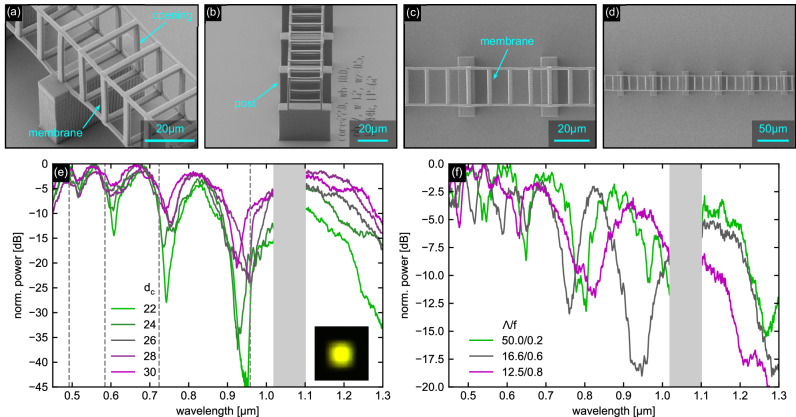
Experimental characterization of Type I Photonic Scaffold. **a**–**d** Selected scanning electron micrographs (SEMs) of a Type I sHCW showing a symmetric membrane arrangement enclosing the central hollow core (*f* = 0.8). **e** Transmission spectra for different core extensions for a constant cladding-openness fraction (color-coded, *Λ* = 14.7 μm, *f* = 0.68, *t* = 1.23 μm, values in μm). The gray dashed lines refer to the resonance wavelength calculated using Eq. (1), including the material dispersion of the polymer^52^. The inset shows the output mode of the waveguide at a wavelength of 575 nm (*d*_*c*_ = 26 μm). **f** Transmission spectra for various pitches (i.e., cladding-openness fraction) at a constant core extension and opening size (color-coded, *d*_*c*_ = 22 μm, *a* = 10 μm, *t* = 1.23 μm). The gray rectangles mask the spectral regime of the pump laser of the light sources, which introduces an artificial peak unrelated to the intrinsic properties of the mHCW. The HCWs shown in (**e**) have a length of 10 mm, while those in (**f**) are 5 mm in length.

**Fig. 4:**
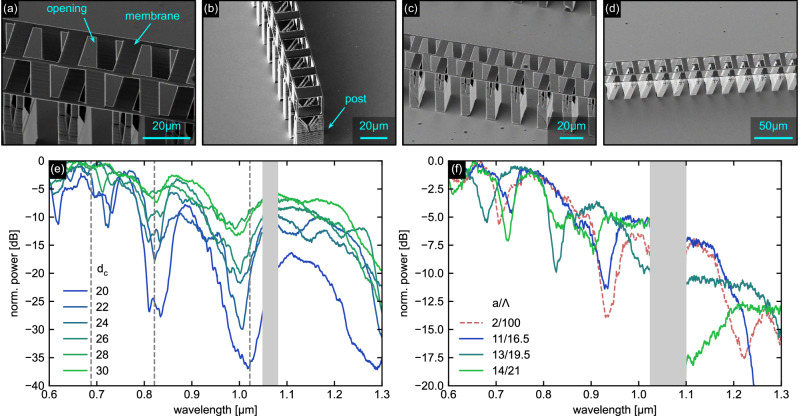
Experimental characterization of Type II Photonic Scaffold. **a**–**d** SEM images of a Type II sHCW featuring a half-period shift between vertical and horizontal openings (*f* = 0.67). **e** Transmission spectra for different core extensions with constant cladding-openness fraction (color-coded, *Λ* = 24 μm, *f* = 0.67, *t* = 1.75 μm, values in μm). Gray dashed lines indicate resonance wavelengths calculated using Eq. (1), accounting for the material dispersion of the polymer^52^. **f** Transmission spectra for varying pitches at constant high cladding-openness fraction (color-coded, *d*_*c*_ = 24 μm, *t* = 1.3 μm, *f* = 0.67, values in μm). The dashed orange curve corresponds to the special case of *f* = 0.02 (*a* = 2 μm), representing an almost fully closed configuration. The gray overlay marks the pump laser region of the light source, which introduces an artificial peak unrelated to the intrinsic sHCW properties. All sHCWs have a length of *L* = 10 mm.

A selection of scanning electron microscope (SEM) images of the fabricated sHCWs is presented in Figs. 3, 4. Both Type I and Type II Photonic Scaffolds feature central cores with extensions (edge-to-edge) ranging from 20 μm to 30 μm and lengths between 5 mm and 30 mm, enclosed by polymer membranes with a thickness of *t* ≈ 1.3 μm. This membrane thickness was selected to maximize the bandwidth of the transmission bands while preserving mechanical stability. The images demonstrate the high structural integrity of both sHCW types, revealing a well-defined periodic sequence of unit cells and the absence of mechanical collapse.

The optical characterization involved measuring the spectral distribution of the fundamental mode across various samples using an unpolarized broadband light source, coupling optics, the sample, and diagnostic tools (see Sec. 4 for details). All spectra were normalized to their respective maxima, with no polarization dependence observed in preliminary measurements.

### Characteristics of Type I sHCW

The analysis begins with investigating the core extension dependence of type I Photonic Scaffold for a constant cladding-openness fraction (*Λ* = 14.7 μm, *f* = 0.68), featuring a symmetric membrane arrangement around the core (Fig. 3(a)). The transmission spectra of the fundamental core mode of selected samples (example of output mode pattern is shown in the inset of Fig. 3e) show alternating regions of high and low transmission from the visible to the near-infrared, confirming that light guidance arises from the anti-resonant effect. Increasing the core extension from *d*_*c*_ = 22 μm (green curve) to *d*_*c*_ = 30 μm preserves the spectral pattern while reducing fringe contrast and enhancing transmission.

To evaluate the impact of introducing openings into the membrane, samples with varying pitches *Λ* (i.e., cladding-openness fractions) were fabricated at constant core extension and opening size and spectroscopically characterized (Fig. 3f). All samples exhibit the characteristic alternating sequence of high and low transmission bands, confirming anti-resonant waveguiding even at an ultrahigh cladding-openness fraction of *f* = 0.8 in agreement with the resonator model (Fig. 2a, b). This is notable given that the membrane extension along the z-direction is only *b* = 2.5 μm, resulting in a structure that is more dominant by air than solid waveguiding material (examples of this structure are shown in Figs. 1, 3a–d). A spectral shift in resonance wavelengths was observed across configurations, attributed to variations in membrane thickness—a parameter that is particularly challenging to control in complex, high-cladding-openness fraction geometries. Despite this, all configurations maintained comparable transmission levels within the transmission bands, which—depending on the application—can be spectrally tuned through geometric adjustments to the sHCW geometry.

### Characteristics of Type II sHCW

The measurement of the core extension dependence of type II Photonic Scaffold reveals a similar trend to its type I counterpart, with increasing transmission and reduced fringe contrast at larger core sizes (Fig. 4e). Notably, the spectral positions of the resonances remain nearly identical across samples, underscoring the high structural quality and reproducibility of the fabricated waveguides.

To assess the impact of the openings on the transmission properties of Type II waveguides, air-dominated structures with constant, ultrahigh cladding-openness fractions but varying opening sizes and pitches were fabricated, characterized, and compared to a reference sample with very small openings (Fig. 4f). Despite spectral shifts in the resonances and transmission bands, which again are attributed to fabrication, all samples exhibit high transmission levels. Notably, the structure with *a* = 11 μm and *Λ* = 16.5 μm (blue curve) reveals an almost identical spectral transmission as the low cladding-openness fraction reference (*a* = 2 μm and *Λ* = 100 μm, orange curve), clearly demonstrating that proper waveguide design enables ultrahigh cladding-openness fraction sHCWs with transmission performance comparable to their closed counterparts even for Photonic Scaffolds geometries that have a higher level of structural complexity.

### Loss properties

**Fig. 5:**
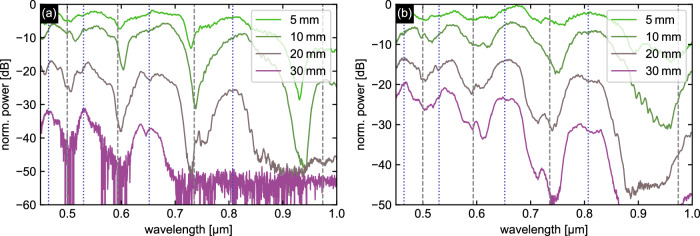
Length-dependent transmission of Type I Photonic Scaffolds. Characterization of the modal losses of two ultrahigh cladding-openness fraction (*f* = 0.68) Type I Photonic Scaffolds based on spectral transmission measurements of samples with different lengths (*a* = 10 μm, *Λ* = 14.7 μm, *t* = 1.25 μm, a *d*_*c*_ = 22 μm, b *d*_*c*_ = 26 μm). Different colors represent varying sample lengths, as indicated in the respective legends. Vertical gray dashed lines indicate the resonances calculated using Eq. (1), and the blue dotted line marks the wavelength at which modal losses were determined (values summarized in Table. 1).

To contextualize the performance of ultrahigh cladding-openness fraction sHCWs, the modal losses of two selected type I Photonic Scaffolds with different core extensions were determined. Transmission measurements were conducted for waveguides of varying lengths, and the transmission/length dependence (in dB) was linearly fitted at selected wavelengths in the transmission bands (see Sec. 4 for details). As expected, the transmission spectra (Fig. 5) show a reduction in transmission with increasing length, attributable to lateral power dissipation caused by leaky mode propagation. Notably, the resonance wavelengths for a given configuration align closely (vertical gray dashed lines in Fig. 5a, b), reflecting the high quality and reproducibility of the fabrication process. The resulting losses at the selected wavelengths (vertical blue dotted lines in Fig. 5) are presented in Tab. 1, with values ranging from 0.5 dB/mm to 0.9 dB/mm for both core extensions considered. For comparison, an additional set of sHCWs with a reduced openness fraction (*f* = 0.20) and a core extent of *d*_*c*_ = 26 μm was fabricated and optically characterized (see Supplementary Note 7), revealing slightly lower loss values for the reduced-openness structure, while all investigated configurations maintain losses below 1 dB/mm within the transmission bands in agreement with simulations presented before. Note that these values are on the same order or even lower than those of comparable on-chip HCWs with closed cladding geometries^19^. As discussed in Supplementary Note 8, transmission-related benchmark quantities have been determined by measuring the optical power at different locations within the experimental configuration. The resulting coupling efficiencies at two selected wavelengths within the corresponding transmission bands are on the order of  ≈ 23–25%. These values can be further improved in future experiments, for example, by fiber interfacing in a V-groove type chip environment^29^ or by directly printing sHCWs on fibers^30^, optimizing mode matching via tailored beam-shaping optics, or by integrating on-chip micro-optics or computationally optimized couplers for enhanced coupling performance. In addition, measurements performed in a water environment for an sHCW with *f* = 0.68 and *d*_*c*_ = 26 μm yield loss values between 0.15 dB/mm and 0.39 dB/mm at visible wavelengths, demonstrating that low-loss guidance is also preserved under liquid conditions. For the longest wavelength considered, increased loss is observed due to the onset of strong NIR absorption of water. Note that, due to the modified spectral response of the sHCW that results from the change in refractive index contrast, different wavelengths are selected for the evaluation of the water case.

**Table 1:** Overview of experimentally determined modal losses for Type I Photonic Scaffolds with two core extents at ultrahigh cladding-openness fraction (*f* = 0.68, (a) *d*_*c*_ = 22 μm, (b) *d*_*c*_ = 26 μm) compared to a structure with moderate openness (*f* = 0.20, *d*_*c*_ = 26 μm) for air and water environment

*f*	environment	*d*_*c*_ [μm]	*λ*_*s*_ [nm]	*γ* [dB/mm]
0.68	air	22	465, 530, 652, 807	0.59, 0.66, 0.86, 0.93
0.68	air	26	465, 530, 652, 807	0.42, 0.56, 0.53, 0.72
0.68	water	26	460, 678, 990	0.15, 0.39, 0.95
0.20	air	26	496, 572, 637, 740	0.27, 0.40, 0.42, 0.46

The loss values are evaluated at selected wavelengths (given in the column labeled *λ*_*s*_) within transmission bands of the sHCW considered; for the waveguide in air with *f* = 0.68, the spectral positions are indicated by vertical blue dotted lines in Fig. 5.

### Application demonstration 1: absorption spectroscopy

**Fig. 6:**
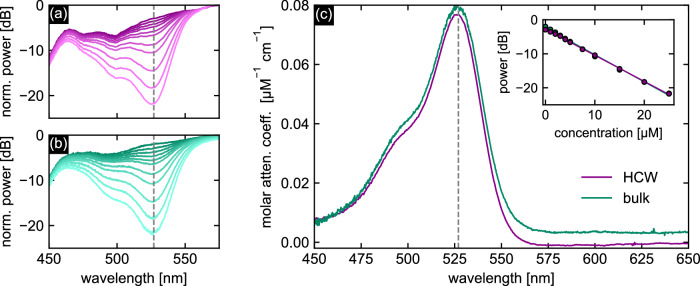
First demonstrated application: Absorption of Rhodamine 6G in the water-filled sHCW. The left column shows the normalized transmission spectra in the visible range for varying R6G concentrations measured using **a** the sHCW (type I, *f* = 0.68) and **b** a bulk reference setup (both 10 mm in length). Concentrations are 0 μM (darkest) to 25 μM (brightest): 0, 1, 2, 3, 4, 5, 7.5, 10, 15, 20, and 25 μM. **c** Molar attenuation coefficient of R6G determined from the sHCW (purple) and the cuvette (dark cyan). Inset: Corresponding calibration curves showing absorbance versus concentration at *λ*_p_ = 527 nm (points: experimental data, line: fits). In all panels, the main absorption peak is indicated by the vertical gray dashed line.

The first demonstration of the application relevance of the Photonic Scaffold approach involves evaluating its performance in integrated spectroscopy. sHCWs with a length of 9.8 mm were immersed in aqueous solutions containing varying concentrations of Rhodamine 6G (R6G), and transmission was measured across the absorption range of the dye (450–650 nm, Fig. 6a; see “Methods”). The obtained spectra show all characteristic absorption features found in reference measurements (Fig. 6b), including the peak at *λ*_p_ = 527 nm. To enable a direct comparison, the molar attenuation coefficient *ε*(*λ*) was calculated for both the sHCW and the reference. The resulting spectra match well (Fig. 6c), confirming the applicability of the sHCW for optofluidic-based integrated absorption spectroscopy. It should be noted that, as confirmed by additional cuvette-based reference measurements, the offset of the molar attenuation coefficient for the reference measurements without waveguide (green curve in Fig. 6c) outside the dye absorption region originates from the chamber itself rather than from the measurement procedure. The corresponding calibration curves showing the absorbance/concentration dependence (inset of Fig. 6c) display linear behavior with identical slopes. The limit of detection (LoD), defined as LoD = 3*σ*_s_/*m* (*σ*_s_: standard deviation of the system), was determined following IUPAC guidelines^31^, with *σ*_s_ evaluated from 1000 blank measurements as described in^16^. The resulting parameters (see Supplementary Table 3) show close agreement between the sHCW and the bulk reference, with molar attenuation coefficients of 7.681 and 7.962 μM^−1^ m^−1^, standard deviations of 0.0194 and 0.0187AU, and corresponding LoDs of 0.0731 μM and 0.0705 μM, respectively. This agreement confirms that the Photonic Scaffold enables the application of the Beer–Lambert law without prior knowledge of the mode profile, in contrast to approaches based on evanescent field interactions. The comparable LoDs further indicate the high spectroscopic performance of the Photonic Scaffold, despite its significantly smaller mode volume compared to the bulk reference measurements.

### Application demonstration 2: dye diffusion

**Fig. 7:**
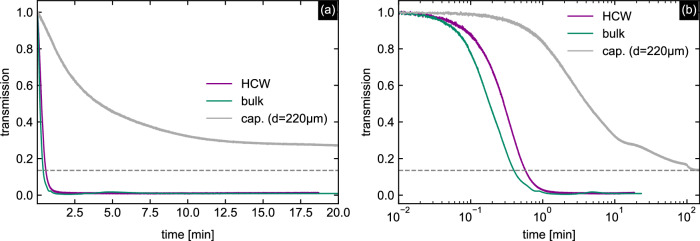
Second demonstrated application: temporal characterization of dye diffusion. Transmission at *λ*_p_ = 527 nm through the water-filled scaffold HCW (purple, core extent 26 μm) as a function of time (**a** linear time scale, **b** logarithmic time scale). For comparison, corresponding measurements are shown for a silica capillary (channel diameter 220 μm, gray) and a measurement without a waveguide, including the objectives (green). In all cases, the light-liquid interaction length is 5 mm. The dashed horizontal lines indicate a power transmission level of $\exp (-2)\approx 13.5\%$.

The second demonstrated application addresses the unique side-wise accessibility of the core of the sHCW, suggesting high performance in diffusion-related scenarios. To show the improvement compared to conventional HCW and bulk configurations, the light transmission through a water-filled sHCW at the absorption peak of R6G (*λ*_p_ = 527 nm) was continuously monitored. When inserting a precise amount of dye into the chamber (see “Methods” section for details), the dye diffusion into the core dynamically reduces the transmission, thus representing an indicator for the diffusion process. Here, the sHCW exhibited a temporal behavior (Fig. 7, purple) nearly identical to that of the waveguide-free configuration (green). To quantify this, the duration of transmission to drop to $\exp (-2)\approx 13.5\%$ (*t*_e_, horizontal gray dashed lines in Fig. 7) was determined in both cases. The results indicate fast diffusion (<1 min) for both configurations, with comparable characteristic times for the sHCW and waveguide-free measurements (sHCW: *t*_e_ = 0.57 min, bulk: *t*_e_ = 0.40 min, Table. 2), indicating that diffusion in the scaffold is only slightly slower compared to the bulk behavior.

The advantageous properties of sHCWs are even more evident when compared to conventional HCWs, which allow access to the core region only through the waveguide ends. In analogous diffusion experiments using a silica capillary with a 220 μm channel diameter—selected as a representative geometry for HCWs in general—a two-order-of-magnitude increase in *t*_e_ was observed (capillary: *t*_e_ = 132.18 min, Tab. 2). It is important to note that we had to chose a significantly larger microchannel diameter (220 μm) than the core of the sHCW (26 μm) to detect any measurable transmission change as diffusion into narrower capillaries is even more limited, and modal losses become exceedingly higher. These experiments therefore demonstrate that dye diffusion into the core region of the Photonic Scaffold closely mirrors that of free diffusion—an important result given the very small transverse extent of the light beam in the sHCW.

**Table 2:** Key parameters relevant for the diffusion experiments, including channel geometries, optical parameters, and diffusion times for three configurations presented in Fig. 7, along with an additional Gaussian beam configuration assuming a beam waist identical to that of the sHCW

Configuration	*t*_e_ [min]	Channel diameter [μm]	Beam div. angle [^∘^]	Input beam waist [μm]	Output beam waist [μm]	*V*_eff_ [nL]
HCW	0.57	26.0	0.00	9.47	9.47	1.41
Capillary	132.18	200.0	0.00	72.85	72.85	83.37
Bulk (no wg)	0.40	–	5.86	0.75	837.42	3671.86
Gaussian beam	–	–	0.76	9.47	66.99	24.40

To quantify the degree of light confinement, we define the effective light-liquid interaction volume as ${V}_{{{{\rm{eff}}}}}=\int_{0}^{L}A(x,y,z)dz$ with the effective area *A*_eff_ (*L*: interaction length, see Supplementary Note 5). Assuming that the beam evolution inside the liquid follows Gaussian beam propagation with the waist located at the input (*z* = 0), the effective volume can be analytically expressed by

7 $${V}_{{{{\rm{eff}}}}}=\pi {w}_{0}^{2}L\left(1+\frac{{L}^{2}}{3{z}_{{\rm{R}}}^{2}}\right)$$

with the beam waist *w*_0_, the interaction length *L*, and the Rayleigh length ${z}_{{{{\rm{R}}}}}=\pi {n}_{{{{\rm{w}}}}}{w}_{0}^{2}/{\lambda }_{0}$ (*n*_w_: refractive index liquid). In the case of a guided mode, *z*_R_ → *∞*, leading to ${V}_{{{{\rm{eff}}}}}=\pi {w}_{0}^{2}L$. In the following, the effective liquid volume of the sHCW (approximated as a cylindrical waveguide) is compared to that of the capillary (Tab. 3), demonstrating that light is confined to a remarkably small volume of approximately 1.4 nL, which is about 60 times smaller than in the capillary (83 nL). The situation of diffraction of an unconfined beam was also considered by measuring the divergence angle of the excitation beam in the absence of the sHCW (beam divergence angle is *θ* = 5.86 °, c.f. Supplementary Note 6), yielding *V*_eff_ = 3671 nL, which is 2600 times larger than that of the sHCW. In addition, a freely diffracting Gaussian beam at the input of the sHCW with a beam waist identical to that of the guided mode (*w*_0_ = 9.47 μm) was also considered. In this case, the resulting output beam waist is 66 μm, corresponding to an effective liquid volume of 24.4 nL, which is 17.3 times larger than that of the sHCW (1.4 nL). These findings underline the unique capability of the sHCW to combine nearly unrestricted analyte diffusion with strong light confinement—a key advantage in diffusion-based sensing.

### Application demonstration 3: single-photon transmission

**Fig. 8:**
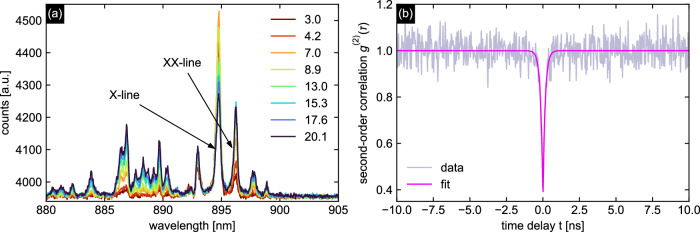
Third demonstrated application: single-photon transmission. **a** Micro-photoluminescence spectra of light from a single quantum dot transmitted through a selected sHCW (*d*_*c*_ = 22 μm, *Λ* = 19.5 μm, *f* = 0.67) at different excitation powers (indicated in the legend, in units of μW). The broad transmission window of the light channel enables the propagation of multiple emission lines, including the exciton (X) and trion (X*) transitions, identified by their characteristic saturation behavior. **b** Second-order autocorrelation measurement of the X line. The fitted exponential decay confirms the single-photon nature of the emission.

The third demonstration of the application relevance of the Photonic Scaffold approach assesses its applicability in photonic quantum technologies by measuring single-photon transmission. Here, emission from a single semiconductor quantum dot (QD) was coupled into a type II sHCW (*d*_*c*_ = 22 μm, *Λ* = 19.5 μm, *f* = 0.67) and analyzed. Note that a Type II sHCW is chosen here because its transmission window matches the required wavelength, avoiding the need for additional sample design. The QDs, embedded in high-brightness micropillar cavities, emitted near the D1 line of Cs at 894 nm^32,33^. A single QD was off-resonantly excited at varying power levels, and micro-photoluminescence spectra at the output of the HCW were recorded (Fig. 8a; see Sec. 4 and Supplementary Note 10). Neutral exciton (X) and a trion (X*) were identified by their characteristic power dependence^34^. To verify single-photon emission, an autocorrelation measurement was performed on the X line (Fig. 8b), yielding a normalized autocorrelation value consistent with single-photon characteristics (see Supplementary Note 11). The preservation of quantum properties, like photon statistics or polarization state, together with a sufficiently broad transmission window, is essential for deploying HCWs in hybrid quantum interfaces. For instance, entangled photons from a cascaded XX–X decay could be efficiently guided through a light cage embedded in a Cs vapor cell functioning as a quantum memory^35^.

## Discussion

When comparing the two types of sHCWs addressed in this study, both exhibit roughly identical transmission levels. However, Type II sHCWs display less regular transmission patterns and a slight reduction in the near-infrared region, indicating that a symmetric membrane arrangement used for Type I sHCW geometry may be more advantageous for efficient light transportation. Note that the investigation of Type II structures serves several objectives: to demonstrate mechanical integrity beyond the symmetric Type I design; to illustrate the capability of 3D nanoprinting to implement 3D architectures with longitudinal variations (axial alternation of openings); and to confirm light guidance in Photonic Scaffolds of higher structural complexity, i.e., with a longitudinally opening distribution that is challenging to realize with other fabrication approaches.

To contextualize the performance of the sHCWs introduced in this study, a comparative overview of key performance metrics for various on-chip HCW concepts from the literature is provided in Supplementary Table 4. Our Type I sHCWs, which incorporate square cores with periodic openings, enable direct side access to the hollow core while maintaining low optical losses and broad spectral operation. The Type I sHCWs, with core dimensions of 22 × 22 μm^2^ and 26 × 26 μm^2^, achieve low losses of 0.4–0.9 dB/mm across the 450–810 nm spectral range. These values are among the lowest reported for anti-resonant on-chip HCWs, demonstrating the effectiveness of our design.

By comparison, conventional membrane-HCWs with smaller cores without openings show higher losses (3.2–3.9 dB/mm and ~10 dB/mm), indicating the efficiency of our open-membrane design. Moreover, the comparison of measured loss values for geometries with different openness fractions (Tab. 1) clearly demonstrates that even ultrahigh openness does not introduce significantly increased attenuation, as losses remain below 1 dB/mm within the transmission bands, thereby confirming the feasibility of the concept of the Photonic Scaffold. While the light cage concept provides side access, it requires a larger core area and more complex fabrication. Our sHCW design uniquely combines an ultrahigh cladding-openness fraction with very low loss and full side access to the core, making Photonic Scaffolds particularly promising for applications requiring both efficient light guidance and direct light-matter interaction with diffusive species.

Within nanoprinted HCWs, modal loss is primarily governed by surface roughness when the microstructured cladding provides sufficient confinement. Notably, the introduction of openings reduces the roughness-induced scattering loss of the mode, effectively counterbalancing leakage-induced losses.

The presented HCW system is well-suited for improving interaction between light and atoms in alkali vapor quantum experiments (e.g., Cs or Rb,^36^), as the diffraction-less propagation of light over millimeter to centimeter distances enhanced the interaction significantly. Prior use of ARROWs in atomic spectroscopy has already highlighted the potential of on-chip HCWs for quantum technologies^14^. The HCW architecture introduced here offers key advantages: (1) maximal core accessibility allowing for efficient atom diffusion into the waveguide, overcoming the slow filling times—often several months—seen in closed systems^23,37^ (see Fig. 1h in ref. ^18^ for an estimate), (2) reduced likelihood of atoms sticking to the walls^38^, further accelerating the filling process, (3) broad transmission window for guiding several wavelengths at the same time, (4) an ultrahigh cladding-openness fraction that minimizes decoherence from atomic spin-flip collisions with the walls and reduces propagation loss arising from surface roughness.

It is important to note that alkali atom diffusion in closed environments deviates from classical or modified Knudsen transport models due to atomic adhesion to the walls^39^. Reference^38^ reports that atoms can remain adsorbed on surfaces for microseconds, likely due to slight diffusion into the glass matrix, explaining the long filling times typically observed in glass cells. The ultrahigh cladding-openness fraction HCW presented here mitigates this issue by drastically reducing wall interaction, thereby shortening diffusion times by orders of magnitude. Based on the analysis shown in ref. ^40^, an estimate of the improvement for Rb filling is provided in Supplementary Note 1. This demonstration of a nanopatterned on-chip HCW in quantum optics employed a nanofilm-protected light cage immersed in cesium vapor to observe electromagnetically induced transparency^18^, indicating the potential for employing other quantum effects such as giant Kerr and single-photon nonlinearities. Note that the sample remained fully functional for over five years without signs of degradation^35^, highlighting the effectiveness of the nanofilm in preventing corrosion^41^. The HCW architecture is also well-suited for cryogenic environments, which is supported by experimental verification provided in Supplementary Note 9, where the spectral transmission of selected sHCWs was measured before and after exposure to liquid nitrogen. The results show only minor amplitude variations while preserving the spectral positions of the resonances, confirming the structural integrity of the Photonic Scaffold under low-temperature conditions.

Another key application of the HCW concept is gas detection, as previously demonstrated using ammonia in 3D nanoprinted light cages and microgap waveguides^18,19^. The ultrahigh cladding-openness fraction design principally enables very rapid diffusion of trace gas species into the core without obstruction from waveguide elements. Beyond tunable diode laser absorption spectroscopy used in the two demonstrations, advanced techniques such as wavelength modulation spectroscopy, lock-in detection, laser power monitoring, and spectrophotometric methods can further be used to enhance sensitivity^42,43^. Relevant sensing examples include ammonia detection in breath for renal diagnostics^44^ and quantification of carbon dioxide and methane for environmental monitoring^45^, both benefiting from the long interaction lengths and low sample volumes offered by compact waveguide platforms.

As demonstrated by the water-based measurements (Tab. 1), the Photonic Scaffold approach offers distinct advantages for optofluidic and analytical applications, where efficient light-matter interaction and rapid analyte exchange are essential. Unlike tubular-type HCWs or ARROWs that suffer from limited core accessibility and slow analyte transport, the sHCW design enables unhindered lateral diffusion into the core, allowing rapid equilibration with the environment—crucial for time-sensitive and dynamic sensing. Simultaneously, it ensures strong light confinement in an exceptionally small interaction volume in the order of nanoliters. It should be noted that in the current experimental implementation, the total sample volume is not minimized, as the open waveguides are immersed in a macroscopic liquid reservoir. Nevertheless, the platform is inherently compatible with microfluidic integration (e.g., fiber interfacing on V-groove enhanced Si-chips^17^), where enclosing the Photonics Scaffold within a microfluidic chamber would enable a substantial reduction of the required sample volume to the microliter or even sub-microliter regime. Note that capillary systems, commonly used in liquid spectroscopy, typically require pressure-driven liquid exchange. In contrast, the openness of the Photonic Scaffold avoids using pressure, preventing mechanical stress and potential structural deformation, while enabling sidewise diffusion of relevant species into the core under controlled conditions. The sHCW further promotes more homogeneous and fast equilibration of the analyte distribution, reduces system complexity by eliminating the need for pumps or fluidic interfaces, and is particularly well suited for applications involving limited sample volumes or sensitive analytes. In biomedicine, the platform can detect specific molecular species^46^ and dynamically monitor nanoscale processes via nanoparticle tracking^22^. Functional surface coatings^47^, including bioreceptors and hydrophobic layers, provide capabilities such as selective binding and clogging prevention. In the field of chemical sensing, the platform can detect industrial compounds, such as alcohols, ketones, and organic acids^48^, support the monitoring of solvent purity in pharmaceutical^49^ and semiconductor processes^50^; and enable the real-time tracking of reactions in microfluidic systems for kinetic and intermediate analysis^51^.

In general, 3D nanoprinting is a versatile technology that enables the fabrication of geometrically complex waveguide structures beyond the capabilities of conventional fiber optical technology. It provides a flexible platform for exploring and benchmarking advanced cladding designs without the complexity and resource demands of fiber fabrication. Notably, the geometry studied here features micrometer-scale periodic variations that are difficult to realize in fiber-based systems.

In summary, we have demonstrated an unexplored class of high-performance membrane-based on-chip HCWs—Photonic Scaffolds—which include cladding-openness fractions up to 80% in the cladding, enabling lateral core access and yielding structures dominated by air rather than solid guiding material. These waveguides show efficient anti-resonant guidance with optical losses comparable to their fully enclosed counterparts, even at the mentioned cladding-openness fractions. The platform was realized through advanced 3D nanoprinting and on-chip integration. Extensive experimental characterization of all relevant parameters, supported by a detailed resonator-based model, confirms the robustness and feasibility of the design. The application potential and advantages were demonstrated in integrated optofluidics and quantum photonics through dye-diffusion experiments, absorption spectroscopy, and single-photon transmission. Due to its combination of efficient light guidance and direct core accessibility, the Photonic Scaffold platform enables exploration of unconventional light-guiding architectures and structural effects beyond the reach of traditional fiber-based systems. With broad applicability across biomedical sensing, integrated analytics, quantum technologies, and environmental monitoring, Photonic Scaffolds are relevant for applications ranging from nano-object detection and photochemical microreactors to vapor-based quantum optics and on-chip gas sensing. Our work expands the design space of hollow-core waveguides, providing a versatile platform for applications in integrated photonics, quantum science, and bioanalytics.

## Methods

### Fabrication

The two types of Photonic Scaffolds were fabricated using two-photon polymerization (2PP) via direct laser writing with a GT2 Nanoscribe system (Nanoscribe GmbH). The photoresist IP-Dip2 was selected for its submicron resolution capability and suitability for high-aspect-ratio structures. Waveguides were printed horizontally in a layer-by-layer manner, using a hatching distance of 150 nm and a slicing distance of 200 nm, resulting in a total fabrication time of approximately 2 h per 10 mm waveguide. After printing, the structures were developed in propylene glycol methyl ether acetate (PGMEA) for 30 min, followed by a final rinse in Novec for 2 min.

### Optical characterization

Transmission measurements were conducted using a broadband supercontinuum light source (450 nm < *λ*_0_ < 2400 nm, SuperK COMPACT, NKT Photonics). To prevent structural damage or overheating from the high-power pump laser, a notch filter with strong absorption at 1064 nm was inserted between the source and sample. Light coupling to the mHCW modes was achieved using an objective lens (20×, NA 0.5), while outcoupled light was collected with an objective lens (10×, NA 0.3). Precise alignment and mode visualization were facilitated by a custom-designed 3D stage and a CCD camera. A flip mirror directed the beam either to the CCD camera for alignment or to a 10× objective, which coupled the light into an optical fiber connected to an optical spectrum analyzer (OSA, AQ6374E, Yokogawa). A pinhole before the collection optics minimized stray light, ensuring selective analysis of the target mode. Transmission spectra were referenced to the source spectrum and individually normalized to their respective maxima. Modal losses were extracted by linearly fitting the transmission data (in dB) as a function of waveguide length at selected wavelengths within a transmission band, with the slope yielding the loss coefficient.

### Absorption spectroscopy

Absorption measurements were conducted using a modified setup to accommodate longer samples. A 10 mm long chamber, housing ~10 mm long waveguides, was employed for these experiments. For the empty chamber reference measurements, the coupling objectives were removed, and a pinhole with a diameter of 0.8 mm was inserted into the input beam path to define the illumination area. Conversely, for measurements involving the sHCW, light was coupled into the waveguides using the objective lens configuration described previously.

Prior to insertion into the fluidic chamber, the silicon chips supporting the sHCW were subjected to plasma treatment for 20 s. This surface activation step ensured complete wetting and prevented the entrapment of air bubbles upon immersion. The concentration-dependent absorption was characterized through a stepwise titration protocol: The chamber was initially filled with 2 mL of deionized water. Target dye concentrations were achieved by sequentially injecting aliquots of a 250 μM R6G stock solution. To ensure solution homogeneity, the liquid was gently mixed via repeated pipette aspiration and dispensing. Particular care was taken during this process to avoid mechanical contact with the chamber walls and to prevent the formation of air bubbles. Transmission spectra were recorded sequentially after each concentration adjustment.

### Dye diffusion

Dye diffusion measurements were performed using a modified version of the transmission setup. To eliminate thermal artifacts arising from infrared absorption, a water-filled cuvette was placed in the beam path in front of the sample chamber. Experiments were conducted in a 5 mm long chamber under three distinct configurations: an empty chamber, a chamber containing the plasma-treated sHCW, and a chamber containing a reference capillary with a 220 μm core diameter. The lengths of the inserted structures were slightly shorter than the chamber width: the sHCW was ≈4.7 mm long due to fabrication margins, while the capillary was cut to ≈4.5 mm to ensure sufficient gaps for fluid filling into the central microchannel. Light was coupled to the samples and subsequently collected using 20 × (NA = 0.5) and 10 × (NA = 0.3) objective lenses, respectively. The collected light was directed to an optical spectrum analyzer (OSA, AQ6374E, Yokogawa) to record the time-dependent transmission.

The diffusion experiments include the following steps: The chamber was initially filled with 300 μL of DI water, ensuring complete immersion of the sHCW or the capillary. Subsequently, 800 μL of an aqueous Rhodamine 6G (R6G, concentration 75 μM) was introduced through the injection channel located near the chamber inlet. This channel was designed to dampen fluidic disturbances, ensuring a stable fluid influx independent of the manual injection procedure. The diffusion process was monitored by recording and evaluating the temporal behavior of the transmitted power at a wavelength of 527 nm with a sampling frequency of 100 Hz.

### Quantum dot experiment

The emission from single QDs was collected and characterized using a cryogenic confocal microscope setup (see Supplementary Fig. 9). The QDs were excited non-resonantly with a 532 nm laser at powers ranging from 3–20 μW, focused through a 100 × objective with NA = 0.8. The sample was kept at 4 K in a closed-cycle cryostat (attoDRY800, Attocube) to ensure stable low-temperature conditions. Fluorescence was collected through the same objective and spectrally filtered using a long-pass filter (850 nm) and a 1 nm bandpass filter to isolate individual lines. The emission was coupled into the mHCW and recollimated using a pair of low-NA 10 × microscope objectives. Spatial filtering via pinholes ensured the selection of only the guided mode from the mHCW. The signal was then coupled into a single-mode fiber and directed either to a spectrometer (SP2500i, Princeton Instruments) with a CCD detector (iDus 420, Oxford Instruments) or to a Hanbury Brown and Twiss interferometer for photon-correlation analysis. The HBT setup used a beam splitter and two superconducting nanowire single-photon detectors (SNSPDs, Eos CS, Single Quantum), with arrival times recorded via a time-correlated single-photon counting module (PicoHarp 300), enabling verification of the single-photon nature of the emission.

## Supplementary information

Supplementary Information

Transparent Peer Review file

## Data Availability

The data supporting this study are publicly available at Zenodo under 10.5281/zenodo.20644151.

## Code availability

The code supporting this study is publicly available at Zenodo under 10.5281/zenodo.17539643.
