# Supplementary material for: Photonic scaffolds as ultrahigh-openness on-chip hollow-core waveguides for quantum photonics and optofluidics
Source: Nat Commun. 2026 Jul 28;17:7563. doi: 10.1038/s41467-026-75873-1 (PMC13415528; doi:10.1038/s41467-026-75873-1)
Supplement: Supplementary file 1 — Supplementary Information [file 41467_2026_75873_MOESM1_ESM.pdf]

Supplementary information for Photonic Scaffolds as Ultrahigh-Openness On-Chip Hollow-Core Waveguides for Quantum Photonics and Optofluidics

Wenqin Huang[1,2†], Diana Pereira[1,3†], Matthias Zeisberger[1], Hala Said[4], Esteban Gómez-López[4], Jun Sun[1,2], Oliver Benson[4], Markus A. Schmidt[1,2,5*]

[1]Fiber Photonics, Leibniz Institute of Photonic Technology, Albert-Einstein-Str. 9, Jena, 07745, Germany.
[2]Abbe Center of Photonics and Faculty of Physics, Friedrich-Schiller-University Jena, Max-Wien-Platz 1, Jena, 07743, Germany.
[3]i3N & Physics Department, University of Aveiro, Campus de Santiago, 3810-193, Aveiro, Portugal, Country.
[4]Department of Physics, Humboldt-Universität zu Berlin, Newtonstraße 15, Berlin, 12489, Germany.
[5]Otto Schott Institute of Materials Research (OSIM), Friedrich Schiller University Jena, Lessingstrasse 12, Jena, 07743, Germany.

*Corresponding author(s). E-mail(s): markus-alexander.schmidt@uni-jena.de;
Contributing authors: wenqin.huang@leibniz-ipht.de; dsap@ua.pt; matthias.zeisberger@leibniz-ipht.de; hala.said@physik.hu-berlin.de; esteban.gomez.lopez@physik.hu-berlin.de; jun.sun@leibniz-ipht.de; oliver.benson@physik.hu-berlin.de;
†These authors contributed equally to this work.

Supplementary Note 1 – Comparison of Filling Time of Alkali Vapor in Waveguides

As shown in Ref. [1], the relative transport time of Rubidium is defined as the time between initial loading and the first detection of more than 2% absorption on the previously unloaded side of the test platform. It is expressed as

$$\Delta t = t_{\text{transport}} - t_{\text{stabilization}} = \eta(L_r/d^2),$$

where L_r is a representative length related to the geometry, η is an experimentally determined constant, and d is the core diameter of the waveguide under consideration. Note that the stabilization time $t_{\text{stabilization}}$ depends on experimental conditions and can range from minutes (as observed in our setup) to several days [1]. For an uncoated glass sample at a temperature of 90°C, $\eta = 24$ μm·days, and with a core diameter of $d = 20$ μm, the relative transport time follows the scaling shown in Supplementary Figure1.

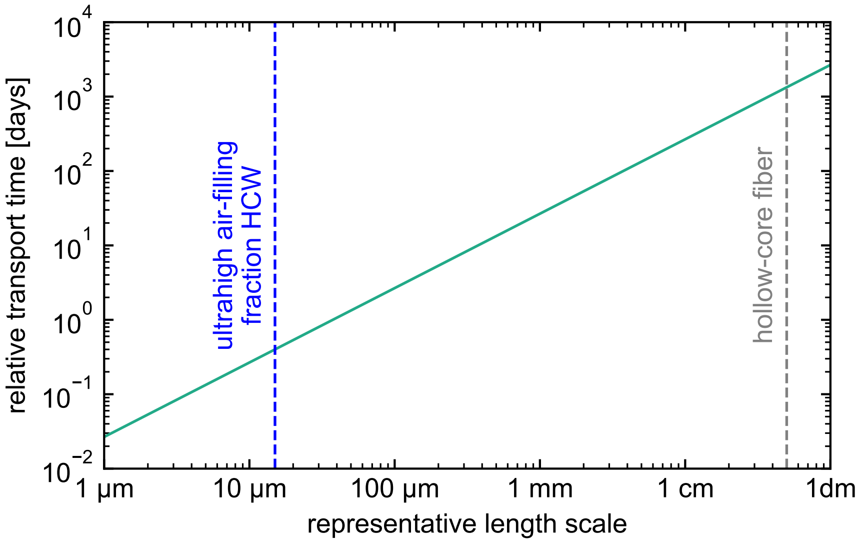

Supplementary Figure 1 Relative transport time versus representative length, calculated by the equation stated in the text. The blue vertical dashed line indicates the Photonic Scaffold ($L_r = 15$ μm ($= d/2$), $\Delta t = 0.3$ days), while the grey curve represents a hollow-core fiber with core access only from the ends ($L_r = 5$ cm, $\Delta t = 300$ days).

To compare the ultrahigh cladding-openness fraction HCWs investigated in this work with commonly used hollow-core fibers in alkali vapor experiments, different representative length scales must be considered (core diameter in both cases: $d = 30$ μm). For the waveguides presented here, sidewise diffusion into the core allows for a representative length of $L_r = 15$ μm, resulting in a relative transport time of $\Delta t =$

0.3 days. In contrast, a typical hollow-core fiber, which is accessible only from the ends, has a representative length of $L_r = 5$ cm, leading to a relative transport time of $\Delta t =$ 300 days, which is 1000 times longer. This long duration severely limits experimental feasibility, as achieving sufficient vapor concentration requires several months [2]. The comparison clearly highlights the advantage of ultrahigh cladding-openness fraction waveguides for vapor-based quantum photonics.

Supplementary Note 2 – Resonator model

As our Photonic Scaffold HCWs lack translational symmetry along the propagation direction, conventional mode solvers—which assume a uniform field variation of the form $\exp(i\beta z)$ and restrict simulation to the transverse (x–y) plane—are not applicable. Instead, we model a finite Photonic Scaffold section of length Λ, corresponding to a single period of the periodic structure, using finite-element modeling (COMSOL Multiphysics). This section is bounded by two fully reflective terminations, forming a resonant cavity whose intrinsic losses (i.e., Q-factor) correlate with the modal attenuation of the actual waveguide (Supplementary Figure 2). The model is implemented in two dimensions, which can be straightforwardly transferred to the full model losses as shown below. Given the symmetry of the fundamental mode with respect to the central axis of the core, only half of the core width ($d_c/2$) is included in the simulation domain. TE and TM modes are treated separately: for the TE mode, perfect magnetic conductor (PMC) boundaries are applied at both the symmetry planes and the terminations; for the TM mode, perfect electric conductor (PEC) boundaries are used. The boundary representing the outer surrounding is implemented as a perfectly matched layer (PML) to suppress artificial reflections from the leaky modes.

2.1 Simulation parameters

In the context of the resonator model, the following simulation parameters were used, consistent with those applied in Fig.4 of the main text:

2.2 Reference simulations to verify the resonator model

To verify the slab-waveguide based resonator model, the results were compared to the spectral distribution of the effective index of the fundamentally leaky mode in a gapless structure, computed using a finite-element solver (Comsol Multiphysics). The resulting distribution (Supplementary Figure 3) shows a sequence of low-loss transmission bands interrupted by sharp resonances. These resonances arise from modal anti-crossings between the central core mode and membrane-supported modes. Notably, the off-resonant regions show extremely low loss values and effective indices in the range of $0.9998 < \mathrm{Re}(n_{\mathrm{eff}}) < 0.9999$, which is very close to $n_c = 1$.

2.3 Estimation of the frequencies of the dissipative band-edge eigenstates

The calculation of the frequencies of the dissipative band-edge eigenstates of the periodic structure by the commercial solver can be significantly accelerated if the

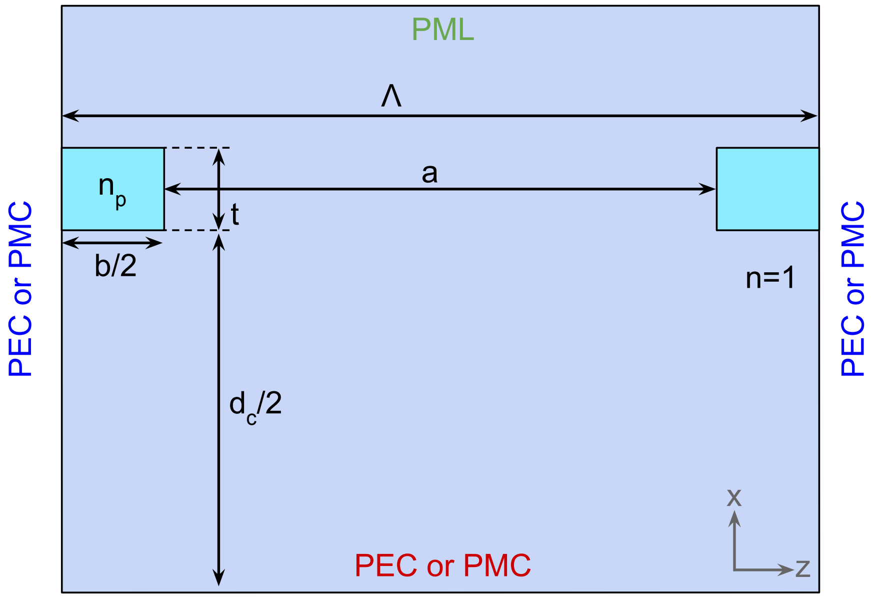

Supplementary Figure 2 Geometry used in the resonator simulations to model the loss behavior of the Photonic Scaffold. Light blue regions indicate air, and cyan regions represent the polymer. The 2D simulations are bounded by appropriate boundary conditions (colored labels) as described in the text.

approximate location of the frequency is known, so that irrelevant solutions can be excluded. To estimate the frequencies, the following box-type resonator model was set up, which is based on the composition of the wave vector.

$$k_0^2 = \beta^2 + \kappa^2, \quad k_0 = \frac{2\pi}{\lambda} = \frac{2\pi\nu}{c}, \quad \kappa = \frac{\pi}{d_c}$$

Using $\beta = 2\pi N/\Lambda$ (N: number of longitudinal modes), the resonance frequencies are given by:

$$\nu = c\sqrt{\left(\frac{N}{\Lambda}\right)^2 + \left(\frac{1}{2d_c}\right)^2}, \quad N = 1, 2, ...$$

In order to search for resonances within the spectral range $\lambda_{\text{min}} < \lambda < \lambda_{\text{max}}$, the corresponding number of longitudinal modes can be approximated by:

$$N_{\text{min}} = \text{int}\left(\frac{\Lambda}{\lambda_{\text{max}}}\right), \quad N_{\text{max}} = \text{int}\left(\frac{\Lambda}{\lambda_{\text{min}}}\right)$$

2.4 Correlation between quality factor and modal losses

To apply the resonator model to the HCW system, the properties of the resonant eigenstates must be linked to the modal characteristics of the waveguide. In this work, this is achieved through the following approach. When the resonator is excited from

Supplementary Table 1 Overview of the parameters used for the resonator-based simulations, including period, filling fraction, membrane thickness, and core diameter, consistent with the configurations analyzed in Fig. 4 of the main text.

Quantity	Symbol	Value
Period	Λ	16.5 μm
Cladding-openness fraction	f	0.0 ... 0.8
Opening length	$a = \Lambda \cdot f$	0.0 ... 13.2 μm
Closed length	$b = \Lambda - a$	16.5 μm ... 3.3 μm
Core diameter	d_c	24 μm
Core refractive index	n_c	1
Membrane thickness	t_m	1.3 μm
Membrane refractive index	n_m	1.5 (constant)

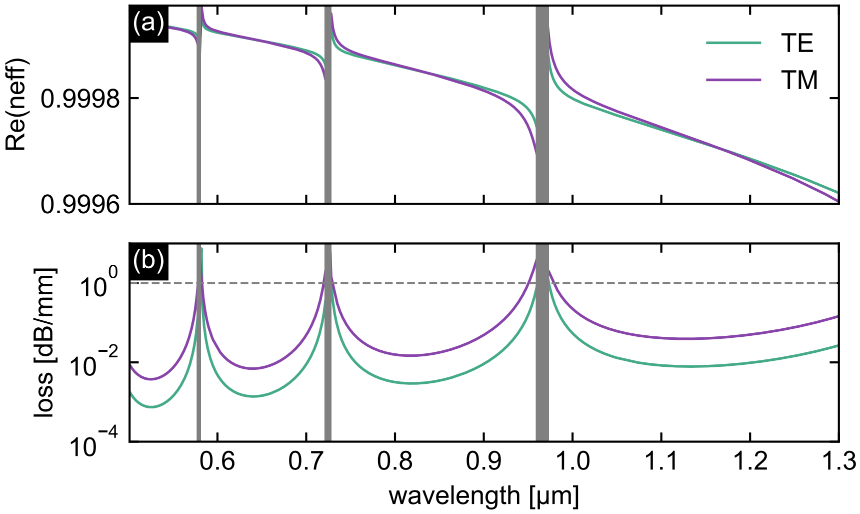

Supplementary Figure 3 Simulated spectral distribution of (a) the real part of the effective index and (b) the modal loss of the fundamentally leaky mode of the square-core HCW section investigated in this study (green: TE polarization, purple: TM polarization). The gray rectangles mask the resonance regions, corresponding to high-loss domains caused by modal anticrossing. The horizontal dashed gray line indicates a loss of 1dB/mm.

one end, it takes a time of $2\Lambda/c$ to fill the waveguide core with forward and backward propagating waves. The energy W stored in the resonator is therefore related to the guided power P_0 by:

$$W = \frac{2P_0\Lambda}{c}$$

A key parameter of any optical resonator is the quality factor Q, defined as the ratio of the stored energy to the energy lost per cycle of oscillation [3]:

$$Q = \frac{2\pi\nu W}{P_L}$$

where P_L is the power lost. If the resonator losses are interpreted as those of a leaky mode propagating through a waveguide of length 2Λ, the modal loss can be expressed as:

$$\gamma_{\text{sw}}\ [\text{dB/mm}] = -\frac{10}{2\Lambda} \lg\left(1 - \frac{P_L}{P_0}\right)$$

Assuming low loss ($P_L/P_0 \ll 1$ on the length Λ), a linear approximation leads to:

$$\gamma_{\text{sw}}\ [\text{dB/mm}] = \frac{27.3}{Q \cdot \lambda\ [\text{mm}]}$$

Thus, the quality factor is directly related to the imaginary part of the effective index:

$$\text{Im}(n_{\text{eff}}) = \frac{1}{2Q}$$

2.5 Correlation between losses of slab waveguide and square-shaped hollow-core waveguide

The presented model is based on calculating the resonator mode of a periodically modulated leaky two-dimensional slab-waveguide system. As demonstrated in one of our previous studies [4], the resulting effective index values are directly linked to those of the square-shaped membrane waveguide. Specifically, as shown in Supplementary Note 2.2 of the Supporting Information of that work, the imaginary part of the fundamental mode of the square-shaped HCW can be related to that of the slab waveguide by:

$$\text{Im}\left(n_{\text{eff}}^{\text{HCW}}\right) = \text{Im}\left(n_{\text{eff}}^{\text{sw,TE}}\right) + \text{Im}\left(n_{\text{eff}}^{\text{sw,TM}}\right) \approx 2 \cdot \text{Im}\left(n_{\text{eff}}^{\text{sw,TE}}\right)$$

The final relation assumes negligible polarization dependence, which is valid in the present case due to the large core size and the symmetric (square) arrangement of the membrane surrounding the core.

Supplementary Note 3 – Design of optofluidic chamber

To ensure stable fluid dynamics during the dye injection process, custom optofluidic chambers were designed and 3D-printed. The lateral sides of the chamber were sealed with microscope cover glasses to provide high-quality optical windows for transmission measurements. As shown in Supplementary Figure 4, the chamber features a specialized flow-dampening inlet (indicated by the angled baffle structure in Supplementary Figure 4(b)). This design serves to mechanically decouple the injection velocity from

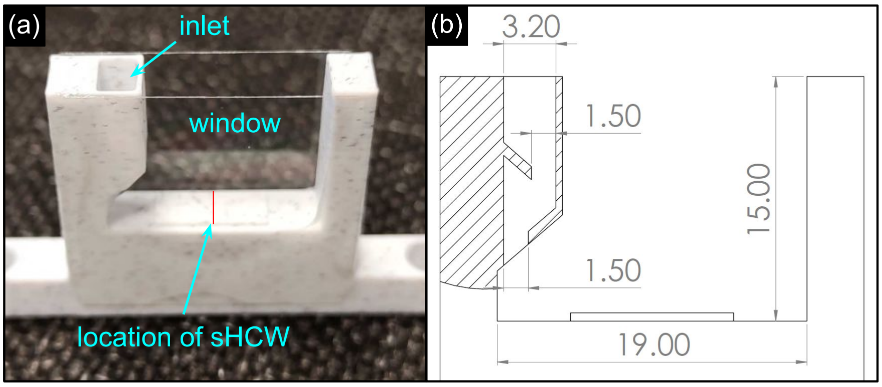

Supplementary Figure 4 Design of the fluidic chamber used for dye diffusion and absorption spectroscopy experiments. (a) Photograph of the fabricated chamber. The red line indicates the location and orientation of the sHCW. **(b)** Cross-sectional technical drawing showing the specific dimensions (in mm) and the internal geometry, highlighting the baffled inlet structure designed to dampen fluidic disturbances.

the fluid flow within the main reservoir. By directing the injected liquid through a baffled path, the structure minimizes fluidic disturbances and prevents the formation of convective jets or air bubbles in the optical measurement zone. This ensures that the observed mixing process is governed primarily by diffusion rather than by the momentum of the manual injection. The chambers were manufactured with widths (optical path lengths) of 5 mm for diffusion experiments and 10 mm for absorption spectroscopy.

Supplementary Note 4 – Correlation between beam waist and core radius

The definition of the effective liquid/light volume includes the Gaussian beam approximation, which requires knowledge on the beam waist w_0, which generally is not identical to the radius of the core section. The following considerations have been conducted to correlated the core radius of a waveguide with cylindrical symmetry to w_0. As first step, we consider a cylindrical waveguide of radius R_c with a radially symmetric field whose intensity profile is approximated by

$$I(r) = I(0)\, J_0^2\left(U \frac{r}{R_c}\right), \qquad 0 \leq r \leq R_c, \tag{1}$$

where J_0 denotes the Bessel function of the first kind, U the modal parameter and r the radial coordinate. In a first order approximation, it can assumed that the field vanishes at the channel wall $r = R_c$, leading to the boundary condition

$$J_0(U) = 0. \tag{2}$$

Hence, U must be a zero of J_0, leading to $U = u_{0m}$ with $m = 1, 2, \ldots$. For the fundamental mode, the modal parameter is given by $U = u_{01} \approx 2.4048256$.

To obtain a correlation between core radius and Gaussian beam waist, the position at which the intensities of the capillary mode drops to a value of $1/e^2$-radius is mapped to a Gaussian waist. Specifically, the intensity distribution of a Gaussian beam is written as

$$I_G(r) = I_G(0) \exp\left(-\frac{2r^2}{w_0^2}\right). \tag{3}$$

By definition, the Gaussian waist parameter w_0 equals the radius at which the intensity drops to $I_G(0)/e^2$:

$$\frac{I_G(w_0)}{I_G(0)} = \exp(-2) = \frac{1}{e^2}. \tag{4}$$

We define a Gaussian-equivalent waist for the capillary mode by matching the same $1/e^2$ condition to the Bessel-type intensity profile. Let r_e denote the radius at which the capillary-mode intensity drops to $I(0)/e^2$:

$$\frac{I(r_e)}{I(0)} = J_0^2\left(U\frac{r_e}{R_c}\right) = \frac{1}{e^2}. \tag{5}$$

In the fundamental lobe ($0 \le r \le R_c$), J_0 is nonnegative, so the equation reduces to

$$J_0\left(U\frac{r_e}{R_c}\right) = e^{-1}. \tag{6}$$

Introducing the normalized radius $\rho_e = r_e/R_c$, we obtain

$$J_0(U\rho_e) = e^{-1}, \qquad 0 < \rho_e < 1. \tag{7}$$

The Gaussian-equivalent waist is then defined as

$$w_0 \equiv r_e = \rho_e\, R_c. \tag{8}$$

As shown before, for the fundamental capillary mode, $U = u_{01}$. Solving Supplementary Equation (7) numerically for ρ_e with $U = u_{01}$ yields

$$\rho_e \approx 0.7285. \tag{9}$$

Using Supplementary Equation (8), the Gaussian-equivalent waist follows as

$$w_0 \approx 0.7285 \cdot R_c \tag{10}$$

Supplementary Note 5 – Effective light–liquid interaction volume

To reveal the degree of light confinement in the three configurations investigated, the effective light-liquid interaction volume V_{eff} was introduced. This parameter refers to

the volume occupied by the light field in absorption-related experiments, acting as an indicator of light confinement. It calculates the area occupied by a Gaussian beam propagating along the positive z-axis starting from the waist at z = 0. Mathematically, the effective light–liquid interaction volume is defined as

$$V_{\text{eff}} = \int_0^L A_{\text{eff}}(z)\, dz, \tag{11}$$

with the effective area

$$A_{\text{eff}}(z) = \frac{\left(\int I(x,y,z)\, dA\right)^2}{\int I^2(x,y,z)\, dA}. \tag{12}$$

For a transverse Gaussian intensity distribution

$$I(r,z) = I_0(z) \exp\left(-\frac{2r^2}{w^2(z)}\right), \tag{13}$$

the effective area is given by

$$A_{\text{eff}}(z) = \pi w^2(z), \tag{14}$$

leading to

$$V_{\text{eff}} = \int_0^L \pi w^2(z)\, dz. \tag{15}$$

Assume a Gaussian beam with waist located at $z = 0$ such that $w(0) = w_0$. For $z > 0$, the intrinsic diffraction leads to

$$w(z) = w_0 \sqrt{1 + \left(\frac{z}{z_R}\right)^2}. \tag{16}$$

Here z_R is the Rayleigh range in the propagation medium. Since diffraction occurs in water with refractive index n_{w}, the Rayleigh range can be written in terms of the vacuum wavelength λ_0 as

$$z_R = \frac{\pi n_{\text{w}} w_0^2}{\lambda_0} \tag{17}$$

Using Supplementary Equations (15) and (16), the effective interaction volume becomes

$$V_{\text{eff}} = \int_0^L \pi w_0^2 \left[1 + \left(\frac{z}{z_R}\right)^2\right] dz = \pi w_0^2 L \left(1 + \frac{L^2}{3z_R^2}\right). \tag{18}$$

If the beam radius is approximately constant over the interaction length such as in a waveguide, $w(z) = w_0$, $z_R \to \infty$ and Supplementary Equation (18) simplifies to

$$V_{\text{eff}} = \pi w_0^2 L. \tag{19}$$

Supplementary Note 6 – Determination of diffraction angle inside liquid chamber

For the calculation of the effective volume V_{eff} in the absence of a waveguide, the diffraction of the light field must be taken into account, characterized by the divergence angle θ. To experimentally determine this parameter, a trial chamber containing only the dye solution without a waveguide was prepared. By launching light at the peak absorption wavelength ($\lambda_{\text{p}} = 527\,\text{nm}$) into the chamber, the beam shape became visible via side-emitted fluorescence. To extract θ, a top-view image was recorded, and the intensity distribution along the x-direction was normalized to its respective maximum at each z-position. At this stage, it is important to note that, since the camera integrates the emitted light along the line of sight, the measured intensity distribution $I(x, z)$ represents the projection of the three-dimensional Gaussian beam profile, with the propagation direction z, the transverse coordinate x, and integration along the imaging axis y.

$$I(x,z) = \int_{-\infty}^{+\infty} I(x,y,z)\,dy \propto \frac{1}{w(z)} \exp\left(-\frac{2x^2}{w(z)^2}\right) \tag{20}$$

As this integration over the y-axis modifies only the prefactor, the exponential term governing the transverse profile remains unchanged, allowing the beam width to be determined from the well-known intensity reduction level $\exp(-2) \approx 0.135$. Therefore, intensity values below 0.135 were cropped and visualized in a different color (Supplementary Figure 5). The divergence angle θ is then defined as the angle between the beam center axis (magenta dashed-dotted line) and the boundary between cropped and uncropped regions (magenta dashed line), yielding $\theta = 5.86°$. Note that the aspect ratio of the image was preserved during processing, so the axis dimensions are irrelevant for angle determination.

Supplementary Note 7 – Impact of cladding-openness fraction on modal losses

To quantitatively evaluate the influence of the cladding-openness fraction on the waveguide losses, an additional set of sHCWs was fabricated and characterized. Specifically, structures with a significantly reduced openness fraction ($f = 0.20$) were compared to the ultrahigh openness structures ($f = 0.68$) investigated in the main text. To ensure a valid and direct comparison, both sets of waveguides were designed to feature identical core dimensions ($d_c \approx 26\,\mu\text{m}$).

Supplementary Figure 6 displays the scanning electron microscope (SEM) images of the fabricated structures, highlighting the distinct structural differences in their cladding regions while maintaining the same central core size. The optical characterization of these devices reveals that the reduced-openness structure exhibits slightly lower loss values compared to the ultrahigh openness design. However, as detailed in the main manuscript (Tab. 1), all investigated configurations maintain modal losses

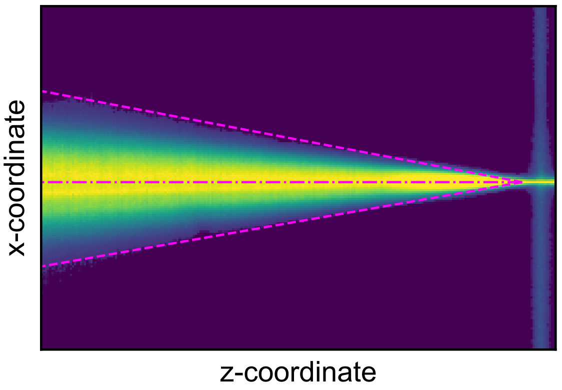

Supplementary Figure 5 Contour plot of the measured spatial distribution of fluorescence near the beam waist, with beam propagation from right to left. At each z-position, intensity values along the x-direction are normalized to the local maximum. Values below $\exp(-2) \approx 0.135$ are cropped and shown in dark blue. Dashed magenta lines mark the cropping threshold, while dotted-dashed lines indicate the beam center. The divergence angle is defined by the angle between these center and boundary lines.

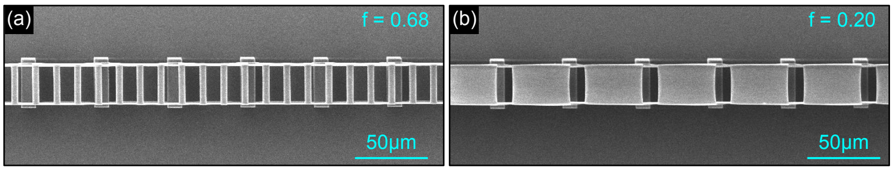

Supplementary Figure 6 Scanning electron microscope (SEM) images of fabricated Type I sHCWs with different cladding-openness fractions used for the loss measurements. (a) Structure with ultrahigh openness ($f = 0.68$), and (b) structure with reduced openness ($f = 0.20$). Both waveguides exhibit comparable core dimensions ($d_c \approx 26\,\mu$m) and are used to investigate the impact of the openness fraction on modal loss. Scale bars: $50\,\mu$m.

below 1 dB/mm within their respective transmission bands. This comparison clearly demonstrates that introducing an ultrahigh openness fraction does not result in significantly increased attenuation, thereby confirming the efficiency and feasibility of the open-membrane Photonic Scaffold design.

Supplementary Note 8 – Determination of coupling efficiency and insertion loss

The coupling efficiency and insertion loss were determined by measuring the optical power at different locations within the experimental configuration (Supplementary Figure 7), enabling a consistent and transparent calculation of the corresponding benchmark quantities.

In the following, the different efficiencies are determined at two selected wavelengths ($\lambda_1 = 546\,$nm, $\lambda_2 = 712\,$nm) within the corresponding transmission band.

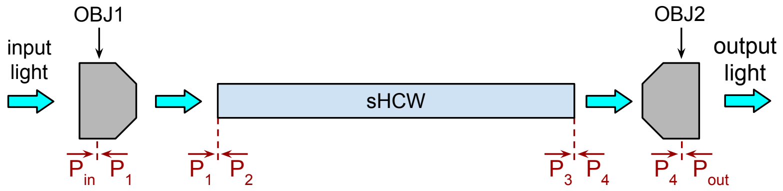

Supplementary Figure 7 Schematic representation of the optical powers at different locations in the experimental configuration (light gray: incoupling/outcoupling lens, light blue: sHCW). The cyan arrows refer to the direction of the light (from left to right). Note that the direction of the red arrows indicates the side of the respective interface (e.g., the arrow for P_1 refers to the power on the left-handed side of the input of the sHCW).

Assuming that all light exiting the waveguide is collected by the output objective, the output power inside the sHCW (P_3) is determined from the measured output power (P_{out}) and the characterized transmission of the objective. This approach assumes that the power at the end facet of the waveguide is identical to the power immediately outside the waveguide, i.e., that interface reflections are negligible ($\eta_{\text{oi}} = P_4/P_3 \approx 1$), which is reasonable given the negligible refractive index discontinuity at the waveguide output. The input power inside the waveguide (P_2) is then calculated from P_3 using the measured propagation loss, which in the present case (sample length 5 mm) are approximately 0.4 dB/mm at 546 nm and 0.62 dB/mm at 712 nm.

Supplementary Table 2 summarizes the resulting efficiencies, including total setup transmission, device transmission, coupling efficiency, and the corresponding insertion losses for both wavelengths.

Supplementary Table 2 Overview of efficiencies and loss metrics used to quantify the optical performance of the experimental configuration for a sample of length 5 mm. Definitions are given in terms of the corresponding power levels at specific locations in the system based on the power definitions introduced in Supplementary Figure 7, and values are evaluated at two selected wavelengths ($\lambda_1 = 546$ nm, $\lambda_2 = 712$ nm).

**name of efficiency**	**symbol**	**definition**	**values @ λ_1 = 546 nm**	**values @ λ_2 = 712 nm**	
total setup	η_s^{tot}	$P_{\text{out}}/P_{\text{in}}$	0.101	0.093	measured
input lens	η_{il}	P_1/P_{in}	0.823	0.867	measured
output lens	η_{ol}	P_{out}/P_4	0.835	0.879	measured
total device	η_d^{tot}	$P_4/P_1 = \eta_s^{\text{tot}}/\eta_{\text{il}}$	0.147	0.121	calculated
output interface	η_{oi}	P_4/P_3	≈ 1	≈ 1	assumed
coupling efficiency	η_c	P_2/P_1	0.234	0.248	calculated
insertion loss (setup)	IL_s	$-10\log_{10}(\eta_s^{\text{tot}})$	$\sim$ 10 dB	$\sim$ 10 dB	calculated
insertion loss (device)	IL_d	$-10\log_{10}(\eta_d^{\text{tot}})$	$\sim$ 8 dB	$\sim$ 9 dB	calculated

The total setup transmission is on the order of ∼10%, corresponding to an insertion loss of approximately 10 dB, which includes all contributions from coupling optics and the waveguide device. By isolating the device contribution, the total device transmission is found to be ≈15% (546 nm) and ≈12% (712 nm), corresponding to insertion losses of ≈8 dB and ≈9 dB, respectively. The resulting coupling efficiency into the waveguide using the current experimental configuration is about ≈23–25%. Approaches to improve this moderate coupling efficiency include fiber interfacing either in a V-groove type chip environment [] or by directly printing sHCWs on fibers [], optimizing mode matching via tailored beam-shaping optics (e.g., aspheric or diffractive elements), or integrating on-chip micro-optics or computationally optimized couplers for enhanced coupling performance.

Supplementary Note 9 – Impact of cryogenic temperatures on the Photonic Scaffold

To assess the robustness of the fabricated Photonic Scaffolds in low-temperature environments, specifically regarding their suitability for cryogenic applications, an additional experimental investigation was conducted. Two types of waveguides with different cladding-openness fractions ($f = 0.20$ and $f = 0.68$) were prepared, and their spectral transmission was initially measured using the setup described in the main manuscript. Subsequently, the samples were fully immersed in liquid nitrogen ($\approx -196°$C, 77 K) for a defined duration of 5 minutes. After returning the structures to ambient conditions, the optical characterization was repeated to evaluate any potential degradation.

The results, presented in Supplementary Figure 8, show that the transmission spectra recorded after exposure to liquid nitrogen (dashed curves) closely match those obtained prior to the exposure (solid curves). This indicates that the severe thermal stress induced by the cryogenic treatment only marginally affects the fabricated structures. For the configuration with a high openness fraction (Supplementary Figure 8(b), $f = 0.68$), slight variations in the transmission amplitude are observed; however, the spectral positions of the resonances remain strictly unchanged. This confirms that the structural integrity and the overall geometry of the waveguide are fully preserved. Overall, these findings demonstrate that exposure to cryogenic environments does not induce optically relevant structural damages to the Photonic Scaffolds, further supporting their integration into low-temperature experimental setups.

Supplementary Note 10 – Experimental setup used for the single-photon experiments

To excite and collect light from a semiconductor quantum dot (QD) embedded in micropillar cavities, an off-resonant laser (532 nm) is focused onto the QD using a 100× microscope objective (NA 0.8, Supplementary Figure 9). The sample is maintained at 4 K in a closed-cycle cryostat to minimize thermal broadening and enable single-photon emission. Photoluminescence from the QD is collected and collimated

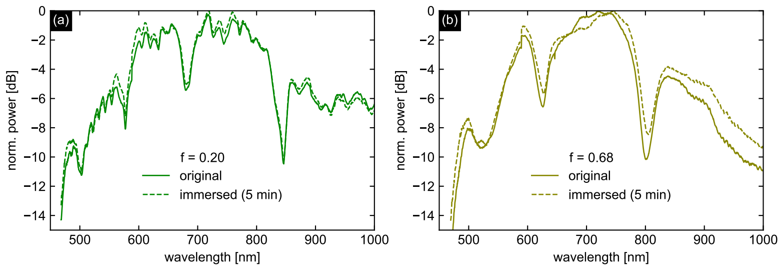

Supplementary Figure 8 Measured spectral transmission of the fundamental core mode for freshly 3D nanoprinted sHCWs (solid curves) compared to the same structures after full immersion in liquid nitrogen for 5 min (dashed curves). Results are shown for two types of structures with different cladding-openness fractions: (a) $f = 0.20$ and (b) $f = 0.68$. The preservation of the resonance positions confirms the structural integrity of the waveguides under cryogenic conditions.

using the same microscope objective, and coupled into the sHCW using a pair of low-NA objectives (10×, NA 0.25). Coupling is optimized using the laser light reflected from the sample and monitored with a CCD camera. After alignment, the excitation laser is removed using a long-pass filter (LP850), leaving primarily QD emission and micropillar cavity resonances. The HCW output is split with a polarizing beam splitter (PBS) and coupled into single-mode fibers for spectral or single-photon detection via a spectrometer or superconducting nanowire single-photon detectors (SNSPDs).

The QD sample consists of In(Ga)As QDs embedded in micropillar cavities grown by molecular beam epitaxy. The structure includes a GaAs/AlAs distributed Bragg reflector (DBR) with 35 layers on the bottom and 15 on top, enclosing a λ-thick cavity layer, where single QDs are self-assembled [5]. Variations in wafer thickness and pillar diameter increase the probability of finding a QD resonant with its micropillar at the desired wavelength (894 for hybrid interfaces with Cs). The spectral properties of the emitted photons and their photon statistics were investigated using the collimated light coming from the HCW. Driving the QDs off-resonantly brings the benefit of a simple excitation laser suppression using a combination of longpass and bandpass filters. One drawback of this excitation method is the phonon-assisted relaxation channels resulting in more emission lines from the QD aside from the neutral exciton (X), biexciton (XX) and trion states (X*), as it can be seen in the spectra shown in the main text Fig.8(a). To identify the transition lines from the investigated QD, the fluorescence intensity (I) as a function of the excitation power (P) is analyzed using a power law fit of the form $I = aP^n$, with a and n as free parameters.The resulting fits allow to associate the 894.6 nm line to the neutral exciton (X) and the 896 nm line to a trion transition (X*), given their different dependency on P. as displayed in Supplementary Figure 10. As can be seen from the spectra, the HCW allows for a wide spectral window to be effectively transmitted. In this case, not only the X and X* lines but all the phonon-assisted processes, and cavity resonances can be transmitted without measurable spectral broadening due to the propagation inside the waveguide.

To verify the single-photon emission of the QD, the second-order autocorrelation function $g^{(2)}(\tau)$ is measured using a Hanbury Brown Twiss (HBT) setup. For a QD, this function can be described with exciton transition linewidth $1/\gamma_x$ as follows [6]:

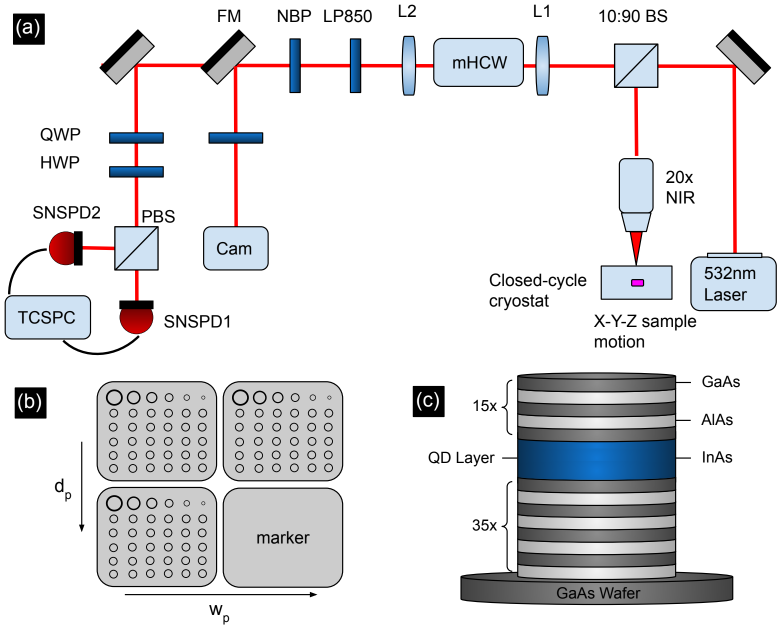

Supplementary Figure 9 (a) Schematic of the setup used to couple light from a single quantum dot (QD) into the HCW, including a 532 nm continuous-wave laser that is embedded in a closed-cycle cryostat at 4 K. The emitted photons are collected and coupled into the light cage via two lenses (L1, L2), filtered using an 850 nm longpass and a 1 nm narrow bandpass filter centered at 894 nm, and directed to the detection system consisting of a polarizing beam splitter (PBS), superconducting nanowire single-photon detectors (SNSPDs), and time-correlated single-photon counting (TCSPC) electronics. (b) Sample layout showing **6** $\times$ **6** micropillar arrays with varying pillar diameter (d_p, vertical direction) and wafer thickness (w_p, horizontal direction). (c) Each micropillar integrates a distributed Bragg reflector (DBR) of alternating GaAs/AlAs layers with an embedded InAs QD in the upper region.

$$g^{(2)}(\tau) = 1 - \left(1 - g^{(2)}(0)\right) e^{-\gamma_x|\tau|}$$

A clear antibunching behavior, expected from a single-photon source, can be observed in Fig.8(b) in the main text. By fitting the raw data, without any background correction, an exciton linewidth $1/\gamma_x \approx 151$ ps and a minimum value of $g^{(2)}(0) = 0.35$ is found, confirming single-photon emission. The reduction of the antibunching dip can be attributed to the presence of photons from other relaxation paths being detected. Therefore, a better spectral filtering of the exciton transition, as well as on-resonant excitation, is required to reduce these multiphoton events.

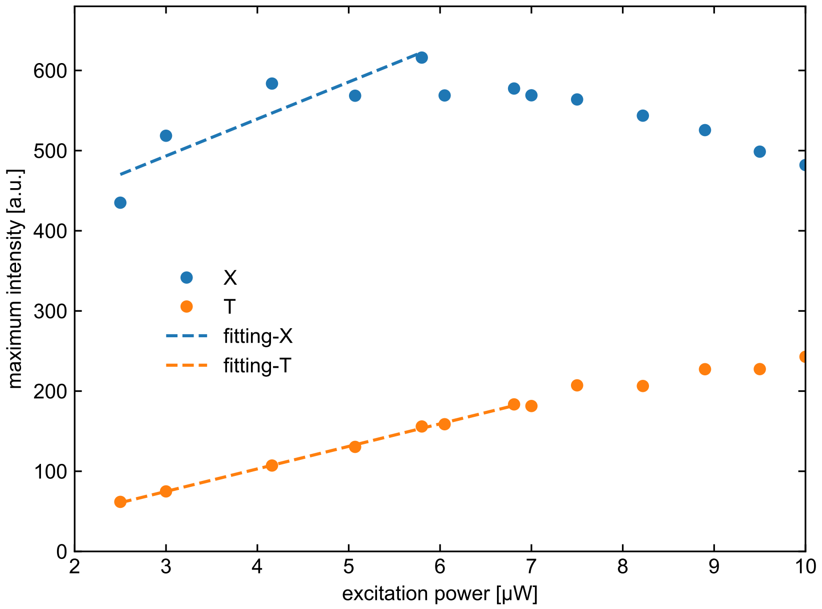

Supplementary Figure 10 Photoluminescence spectra of the exciton (X-line, blue dots) and trion (T, orange dots) emission lines as a function of excitation power. The dashed lines represent fits to the data points in the low-power regime.

Supplementary Note 11 – Identification of emission lines

To analyze the photoluminescence spectra (Fig.8 in the main text), each emission line was fitted with a Lorentzian function to determine its peak position and intensity. Two emission peaks were identified for each excitation power, corresponding to the exciton (X) and trion (X*) transitions. Prior to fitting, background correction was performed by subtracting the counts generated by the dark current of the CCD detector in the spectrometer.

Supplementary Note 12 – Spectroscopic performance parameters of the sHCW and bulk reference

To provide a comprehensive quantitative assessment of the application performance of the Photonic Scaffold approach in integrated optofluidic spectroscopy, this section summarizes the key spectroscopic parameters determined from the absorption measurements. sHCWs with a length of 9.8 mm were immersed in aqueous solutions containing varying concentrations of Rhodamine 6G (R6G), and their performance was evaluated against a macro-scale bulk reference configuration.

The evaluation includes the experimentally determined molar attenuation coefficient $\varepsilon(\lambda)$ at the peak absorption wavelength ($\lambda_p = 527\,\text{nm}$) and the system standard deviation σ_s, which was rigorously calculated from 1000 blank measurements. Following the IUPAC guidelines, the limit of detection (LoD) was defined as $\text{LoD} = 3\sigma_s/m$, where m denotes the slope of the corresponding calibration curve. The extracted performance metrics for both configurations are detailed in Supplementary Table 3. The high degree of correspondence between the parameters underlines that the Beer-Lambert law remains fully valid within the sHCW architecture without necessitating prior knowledge of the local mode profile, while significantly minimizing the required analyte and mode volume.

Supplementary Table 3 Comparison of spectroscopic performance parameters between the macro-scale bulk reference and the on-chip sHCW configuration.

**Configuration**	Molar atten. coeff. [$\mu\text{M}^{-1}\text{m}^{-1}$]	Standard deviation σ_s [AU]	LoD [μM]
Bulk	7.962	0.0187	0.0705
sHCW	7.681	0.0194	0.0731

Supplementary Note 13 – Comparison with state-of-the-art on-chip hollow-core waveguides

To contextualize the optical performance of the Photonic Scaffolds investigated in this work, this section provides a comparative overview of key performance metrics for various state-of-the-art on-chip hollow-core waveguides (HCWs) reported in the literature. Supplementary Table 4 summarizes the guiding mechanism, core geometry and area, optical losses, spectral operating range, and the capability for direct side access to the core. This comparison highlights that the sHCW design uniquely combines an ultrahigh cladding-openness fraction with very low attenuation and broad spectral operation, standing out among existing integrated HCW architectures.

Supplementary Table 4 Comparison of key performance indicators for the two type I sHCWs characterized in this work (Fig. 5) alongside state-of-the-art on-chip HCWs from the literature, including guiding mechanism, core geometry and area, optical losses, spectral operating range, and side access capability.

Study	Guiding mechanism	Shape	Side access	Core extent [µm]	Core area [µm]	Losses [dB/mm]	Spectral range [nm]	Ref.
Type I Photonic Scaffold	AR	square with opening	yes, very high	22 × 22	484	0.6–0.9	450–810	this work
Type I Photonics Scaffold	AR	square with opening	yes, very high	26 × 26	676	0.4–0.7	540–810	this work
membrane HCW	AR	square	no	8.0 × 7.2	57.6	3.2–3.9	450–750	[7]
membrane HCW	AR	square	no	7.4 × 7.4	54.8	∼10	450–750	[8]
microgap HCW	AR	square	partially	20 × 20	400	0.38–1.25	420–1400	[4]
ARROW	multilayer	square	no	3.5 × 24	84	2.8	785	[9]
high index grating	sub-λ grating	square	no	9 × 43	387	0.037	1480–1579	[10]
light cage	AR	hexagonal	yes	24 × 28	680	0.5–1	450–750	[11]

References

[1] Giraud-Carrier, M., Decker, T., McClellan, J., Bennett, L., Hawkins, A., Black, J., Almquist, S., Schmidt, H.: Temperature and wall coating dependence of alkali vapor transport speed in micron-scale capillaries. Journal of Vacuum Science & Technology A **35**(3), 031602 (2017) https://doi.org/10.1116/1.4978888

[2] Epple, G., Kleinbach, K.S., Euser, T.G., Joly, N.Y., Pfau, T., Russell, P.S.J., Löw, R.: Rydberg atoms in hollow-core photonic crystal fibres. Nature Communications **5**(1), 4132 (2014) https://doi.org/10.1038/ncomms5132

[3] Jackson, J.D.: Classical Electrodynamics, 3rd edn. John Wiley & Sons, Nashville, TN (1998)

[4] Bürger, J., Schalles, V., Kim, J., Jang, B., Zeisberger, M., Gargiulo, J., S. Menezes, L., Schmidt, M.A., Maier, S.A.: 3d-nanoprinted antiresonant hollow-core microgap waveguide: An on-chip platform for integrated photonic devices and sensors. ACS Photonics **9**(9), 3012–3024 (2022) https://doi.org/10.1021/acsphotonics.2c00725

[5] Unsleber, S., Schneider, C., Maier, S., He, Y.-M., Gerhardt, S., Lu, C.-Y., Pan, J.-W., Kamp, M., Höfling, S.: Deterministic generation of bright single resonance fluorescence photons from a purcell-enhanced quantum dot-micropillar system. Opt. Express **23**(26), 32977–32985 (2015) https://doi.org/10.1364/OE.23.032977

[6] Robert, I., Moreau, E., Gérard, J.M., Abram, I.: Towards a single-mode single photon source based on single quantum dots. Journal of Luminescence **94-95**, 797–803 (2001) https://doi.org/10.1016/S0022-2313(01)00363-5 . International Conference on Dynamical Processes in Excited States of Solids

[7] Pereira, D., Wieduwilt, T., Hauswald, W., Zeisberger, M., Ferreira, M.S., Schmidt, M.A.: 3d nanoprinted fiber-interfaced hollow-core waveguides for high-accuracy nanoparticle tracking analysis. Light: Science & Applications **14**(1), 197 (2025) https://doi.org/10.1038/s41377-025-01827-9

[8] Pereira, D., Ferreira, M.S., Zeisberger, M., Schmidt, M.A.: Spatially controlled phase modulation - selective higher-order mode excitation in 3d nanoprinted on-chip hollow-core waveguides. ACS Photonics **11**(8), 3178–3186 (2024) https://doi.org/10.1021/acsphotonics.4c00524

[9] Yin, D., Schmidt, H., Barber, J.P., Hawkins, A.R.: Integrated arrow waveguides with hollow cores. Opt. Express **12**(12), 2710–2715 (2004) https://doi.org/10.1364/OPEX.12.002710

[10] Yang, W., Ferrara, J., Grutter, K., Yeh, A., Chase, C., Yue, Y., Willner, A.E., Wu, M.C., Chang-Hasnain, C.J.: Low loss hollow-core waveguide on a silicon substrate. Nanophotonics **1**(1), 23–29 (2012) https://doi.org/10.1515/nanoph-2012-0003

[11] Bürger, J., Kim, J., Jang, B., Gargiulo, J., Schmidt, M.A., Maier, S.A.: Ultrahigh-aspect-ratio light cages: fabrication limits and tolerances of free-standing 3d nanoprinted waveguides. Opt. Mater. Express **11**(4), 1046–1057 (2021) https://doi.org/10.1364/OME.419398
