# Supplementary material for: Photonic scaffolds as ultrahigh-openness on-chip hollow-core waveguides for quantum photonics and optofluidics
Source: Nat Commun. 2026 Jul 28;17:7563. doi: 10.1038/s41467-026-75873-1 (PMC13415528; doi:10.1038/s41467-026-75873-1)
Supplement: Supplementary file 2 — Transparent Peer Review file [file 41467_2026_75873_MOESM2_ESM.pdf]

nature portfolio

Peer Review File

Photonic Scaffolds: On-Chip Hollow-Core Waveguides with Ultrahigh Openness for Integrated Quantum Optics and Optofluidics

Corresponding Author: Professor Markus Alexander Schmidt

This file contains all reviewer reports in order by version, followed by all author rebuttals in order by version.

Version 2:

Reviewer comments:

Reviewer #1

(Remarks to the Author)
This interesting manuscript introduces an improved “scaffold-type” on-chip hollow-core waveguide design, fabricated by direct-write techniques. The work builds on previous results on square-core membrane waveguides by the same group, published in ACS nano in 2022 [Ref 19]. The key novelty of the improved design is that the sidewalls of the 3D-printed waveguide structure contain large gaps, making it much easier for gases and or liquids to access the waveguide sideways. In comparison, the previous design [Ref 19] was based on short sections of rectangular tubes, separated by small gaps.

At first glance, the improvement compared to the earlier work appears somewhat incremental. However, the authors offer an excellent description of the guidance mechanism of their scaffold waveguide, which is explained by a combination of commonly used anti-resonant reflection effects, and the formation of dissipative Bloch modes in the structure by the periodic nature of the transverse scaffolds that comprise the waveguide. While the use of such periodic structures leads to stop bands, and therefore reflections, the variations in effective refractive index caused by the scaffold design are so small that the losses due to reflection remain below 4% over tens of mm propagation length. The authors demonstrate, by simulations, that their open scaffold design can indeed act as a hollow-core waveguide with losses of less than 1dB/mm, which is relatively low for on-chip waveguides. I find this approach elegant and the results quite surprising.
The authors fabricate two sets of slightly different scaffold-waveguide designs, with a range of different lengths. The transmission spectra of the waveguides are measured as well as, importantly, their loss spectra (Fig 6). The results are in fairly good agreement with the predicted values, with experimental losses remaining below 1dB/mm.

Two test experiments are performed with the produced waveguides. In the first, the on-chip waveguide is placed at the bottom of a liquid reservoir, in which a droplet of dye is released at some distance away from the waveguide. The absorption through the waveguide is measured as a function of time, to as to measure the diffusion of the dye.
The scaffold-waveguide results are in good agreement with the free-space beam experiment (Fig 7), apart from a factor 1.5 times slower response time (Fig 8). This result demonstrates that the open waveguide structure allows an almost instantaneous detection of the surrounding concentration, due to the short diffusion distances involved.

The second example is the coupling of emission from a quantum dot a single-photon source to a scaffold waveguide. The light is guided to a standard Hanbury Brown Twiss setup and antibunching behaviour shows that the emission is indeed in the single-photon domain. The main point of this experiment is that it shows that polarisation state and photon statistics are preserved during propagation through the waveguide, which is important for future quantum applications of the work.

The discussion section compared the main properties of the scaffold waveguides to other on-chip waveguides (table 4), and then discusses a range of potential application areas of the work.

The text is well-written, provides enough detail about the methods used, and graphs are clear. The analysis is solid, and the data appear to support the conclusions. Overall, this is an interesting paper. The scaffold waveguide concept is quite novel and clearly facilitates the transverse diffusion of samples into the waveguide core region, as is nicely demonstrated using dye in water. The largest impact of this work, in my opinion, would be in quantum optics where alkali vapours such as Cs or Rb, can diffuse into the scaffold-waveguide much more rapidly than in conventional hollow-core fibres. This potential

application area makes the work of interest to the Nature Communications readership.

Before I can recommend publication of this Manuscript, however, I would like the authors to address the following comments:

Comments:

1. The authors compare diffusion into their scaffold waveguide to diffusion into a hollow capillary. Obviously, this results in very significant differences in diffusion time. In liquid spectroscopy, capillaries would normally be filled under pressure and then rely on transverse diffusion over a much shorter distance across the core, and therefore, much faster response times.

2. How robust is the scaffold structure against transverse fluid flow? Have the authors tested the maximum transverse flow velocity it can withstand?
The authors also state that the scaffold waveguides can withstand cryogenic temperatures, but no supporting data are provided. Have they tried cooling down the structures, and do they survive the thermal stress involved?

3. Table 3: The comparison of the mode volume of the scaffold waveguide and the "Bulk"/diverging beam is quite biased by the beam waist parameter chosen for the free-space beam. The authors choose an input beam waist of 0.75 um, which results in a mode volume of 3671 nL (compared to 1.41 nL for the scaffold waveguide with a beam waist of 9.47 um). In their table, the authors should also include a row showing the effective volume of a free-space beam with the same waist diameter as the scaffold waveguide (9.47 um). A quick check of Equation 7 suggests that this would result in an effective volume for the free-space beam that is two orders of magnitude smaller than for the tightly-focused beam currently shown in table 3. Note that the value for the less-diverging beam would still be 30-40 times larger than that of the scaffold waveguide, but it would, in my opinion, be a fairer way to compare its performance to that in a bulk experiment.

4. What are the losses in liquid-immersed waveguides? Were these measured/estimated, and are they larger/smaller than the air-guided waveguides?

5. In the loss spectra in Fig 7, why is there an absorbance offset in the bulk absorption spectra in the long-wavelength region? Was this offset reproducible, or is it due to a baseline drift?

6. On page 12 (Figure 8) the authors claim that the temporal behaviour of the scaffold waveguide is "nearly identical" to that of the bulk experiment. Considering the logarithmic time axis, however, there is still a 1.5 times difference in the time it takes for the absorbance to reach 50% of its peak value, which I would not describe as nearly identical. Please rephrase.

7. On page 14, the authors write: "By comparison, conventional membrane-HCWs with smaller cores without openings show higher losses (3.2–3.9 dB/mm and ~10 dB/mm), highlighting the efficiency of our open-membrane design". Because the membrane - HCWs features smaller cores, which always result in higher waveguide losses, this result does, in my opinion, not highlight the "efficiency of the open-membrane design". To support this statement, losses of open and closed membrane waveguides with the same core diameter should be compared. Please include such data or rephrase the statement. Please include the core diameter of the (closed) membrane waveguide in the manuscript.

8. On page 16, the authors write:" Simultaneously, it ensures strong light confinement in an exceptionally small interaction volume, enabling nanoliter-scale, high-sensitivity detection with minimal sample consumption."
While I agree that the interaction volume is very small, it is not correct to say that their current approach minimises sample consumption. In their liquid experiments, the open waveguides are immersed in 1 mL of sample, of which only a small fraction enters the waveguide core. If the authors were to minimise their sample volume, the open waveguide would need to be enclosed within a slightly larger microfluidic channel. The resulting structure would then look very similar to optofluidic hollow core fibres. Please comment on this in the manuscript.

(Remarks on code availability)

Reviewer #2

(Remarks to the Author)
The authors present a novel geometry for an on-chip hollow-core waveguide. Hollow-core waveguides have been around for a while, and this group are leading the research. The geometry they present here is truly novel and I am impressed by the high performance it exhibits despite the very open structure. I agree that the open structure is very exciting, as it gives easy access to the analyte and changes within it; the diffusion work shown in fig. 8 clearly highlights this capability.

I only have some minor comments:
1. I do not understand why they separate between type I and type II. Clearly, there are thousands of variants possible with this geometry. Reading the paper, I find the discussion of the two types distracting, and I don't really understand why the two types are needed. In table 4, I assume there is a typo, as the first two rows both refer to type I; should this be type I (first row) and type II (second row)? If so, the performance is very similar, so again, why do we need to see the difference? In conclusion: If you want to keep the two types, make the distinction very clear: "Type 1 has these unique properties, while type II offers a different trade-off leading to a different set of unique properties." As it stands, type 2 is only introduced to highlight the advantage of the symmetry of type I. If that's all, please move type II to the SI in its entirety.

2. Can you please specifiy the coupling efficiency/insertion loss? I assume efficiency is high, but that would be good to confirm.

Overall, a well-written paper. I apprecaite the clarity of description without the overuse of superlatives one often sees these days.

(Remarks on code availability)

Version 3:

Reviewer comments:

Reviewer #1

(Remarks to the Author)
I thank the authors for submitting a thoroughly revised version of their manuscript. I particularly appreciate the additional experiments conducted. All my comments have been adequately addressed, and I am happy to recommend this Manuscript for publication in Nature Comms.

(Remarks on code availability)

Reviewer #2

(Remarks to the Author)
I thank the authors for their well-considered response and the additional work they have conducted to address mine and the other reviewer's concerns. On the basis of these revisions, I fully support publication.

(Remarks on code availability)

Open Access This Peer Review File is licensed under a Creative Commons Attribution 4.0 International License, which permits use, sharing, adaptation, distribution and reproduction in any medium or format, as long as you give appropriate credit to the original author(s) and the source, provide a link to the Creative Commons license, and indicate if changes were made.
In cases where reviewers are anonymous, credit should be given to 'Anonymous Referee' and the source.
The images or other third party material in this Peer Review File are included in the article’s Creative Commons license, unless indicated otherwise in a credit line to the material. If material is not included in the article’s Creative Commons license and your intended use is not permitted by statutory regulation or exceeds the permitted use, you will need to obtain

permission directly from the copyright holder.
To view a copy of this license, visit https://creativecommons.org/licenses/by/4.0/

Response letter for our manuscript "*Photonic Scaffolds: On-Chip Hollow-Core Waveguides with Ultrahigh Openness for Integrated Quantum Optics and Optofluidics*" (ID NCOMMS-25-89336B-Z)

Dear Reviewers,

Please find attached the revised version of our manuscript entitled "*Photonic Scaffolds: On-Chip Hollow-Core Waveguides with Ultrahigh Openness for Integrated Quantum Optics and Optofluidics*" by Wenqin Huang, Diana Pereira, Matthias Zeisberger, Hala Said, Esteban Gomez-Lopez, Jun Sun, Oliver Benson and Markus A. Schmidt. We are grateful to the Reviewers for the feedback and for raising important points, which have helped us to improve the manuscript.

Before going into details, we would like to note that, in response to the comments, the experimental part of the study has been substantially extended by five additional measurements, some of which required additional sample fabrication:

I. loss measurements in air and aqueous environments
II. loss measurements on a sample with smaller openings to assess the impact of the openness fraction on modal loss,
III. immersion of freshly prepared and characterized waveguides in a liquid nitrogen environment to evaluate the impact of cryogenic conditions
IV. concentration-dependent absorption measurements of the bulk chamber without the chamber; and
V. coupling efficiency measurements at two selected wavelengths.

In light of this, the Supplementary Information has been significantly expanded to provide detailed experimental and analytical descriptions of the new results, and the main text has been revised and extended accordingly.

In the following, the Reviewer comments are given in black. Please find our responses in blue and the revised text in green (changes are highlighted in **green bold**). Please note that all changes (except minor spelling corrections) are detailed in this response letter, including the locations where they appear in the manuscript.

We take the opportunity to thank the Reviewers once again for their constructive comments and suggestions, which helped us to substantially improve the quality of our manuscript. Having responded to their queries in detail, we look forward to your response.

Sincerely,

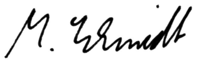

Markus A. Schmidt

Response to comments of Reviewer #1

This interesting manuscript introduces an improved “scaffold-type” on-chip hollow-core waveguide design, fabricated by direct-write techniques. The work builds on previous results on square-core membrane waveguides by the same group, published in ACS nano in 2022 [Ref 19]. The key novelty of the improved design is that the sidewalls of the 3D-printed waveguide structure contain large gaps, making it much easier for gases and or liquids to access the waveguide sideways. In comparison, the previous design [Ref 19] was based on short sections of rectangular tubes, separated by small gaps. At first glance, the improvement compared to the earlier work appears somewhat incremental. However, the authors offer an excellent description of the guidance mechanism of their scaffold waveguide, which is explained by a combination of commonly used anti-resonant reflection effects, and the formation of dissipative Bloch modes in the structure by the periodic nature of the transverse scaffolds that comprise the waveguide. While the use of such periodic structures leads to stop bands, and therefore reflections, the variations in effective refractive index caused by the scaffold design are so small that the losses due to reflection remain below 4% over tens of mm propagation length. The authors demonstrate, by simulations, that their open scaffold design can indeed act as a hollow-core waveguide with losses of less than 1dB/mm, which is relatively low for on-chip waveguides. I find this approach elegant and the results quite surprising.

The authors fabricate two sets of slightly different scaffold-waveguide designs, with a range of different lengths. The transmission spectra of the waveguides are measured as well as, importantly, their loss spectra (Fig 6). The results are in fairly good agreement with the predicted values, with experimental losses remaining below 1dB/mm. Two test experiments are performed with the produced waveguides. In the first, the on-chip waveguide is placed at the bottom of a liquid reservoir, in which a droplet of dye is released at some distance away from the waveguide. The absorption through the waveguide is measured as a function of time, as to measure the diffusion of the dye. The scaffold-waveguide results are in good agreement with the free-space beam experiment (Fig 7), apart from a factor 1.5 times slower response time (Fig 8). This result demonstrates that the open waveguide structure allows an almost instantaneous detection of the surrounding concentration, due to the short diffusion distances involved.

The second example is the coupling of emission from a quantum dot, a single-photon source to a scaffold waveguide. The light is guided to a standard Hanbury Brown Twiss setup and antibunching behaviour shows that the emission is indeed in the single-photon domain. The main point of this experiment is that it shows that polarisation state and photon statistics are preserved during propagation through the waveguide, which is important for future quantum applications of the work.

The discussion section compares the main properties of the scaffold waveguides to other on-chip waveguides (table 4), and then discusses a range of potential application areas of the work.

The text is well-written, provides enough detail about the methods used, and graphs are clear. The analysis is solid, and the data appear to support the conclusions. Overall, this is an interesting paper. The scaffold waveguide concept is quite novel and clearly facilitates the transverse diffusion of samples into the waveguide core region, as is nicely demonstrated using dye in water. The largest impact of this work, in my opinion, would be in quantum optics where alkali vapours such as Cs or Rb, can diffuse into the scaffold-waveguide much more rapidly than in conventional hollow-core fibres. This potential application area makes

the work of interest to the Nature Communications readership.

We thank the Reviewer for the careful reading of the manuscript and for the valuable and positive feedback. In the following, all points are addressed in a detailed, point-by-point manner, and the corresponding revisions in the manuscript are indicated where appropriate.

Before I can recommend publication of this Manuscript, however, I would like the authors to address the following comments:

Comments:

R1.1: The authors compare diffusion into their scaffold waveguide to diffusion into a hollow capillary. Obviously, this results in very significant differences in diffusion time. In liquid spectroscopy, capillaries would normally be filled under pressure and then rely on transverse diffusion over a much shorter distance across the core, and therefore, much faster response times.

We thank the Reviewer for this important comment and for highlighting the role of pressure-driven filling in conventional capillary-based systems. We agree that, in typical liquid spectroscopy using capillaries or fibers with thick claddings, pressure-assisted filling can be employed and enables fast response times. However, several aspects should be considered in the context of the present work:

A. Structural integrity of the waveguide: For the on-chip hollow-core waveguides investigated here, the situation is fundamentally different. Due to the thin membrane-based cladding, the application of elevated pressure can lead to mechanical deformation or damage of the structure, making pressure-driven filling impractical. In addition, pressure-induced deformation may alter the membrane thickness, resulting in spectral shifts or narrowing of the transmission bands.

B. Analysis of dynamically changing liquid environments: The open scaffold geometry enables direct access to the core, which is particularly advantageous when studying the response of a selected species to externally induced changes in the liquid environment. For example, in nanoparticle tracking analysis (NTA), an ensemble of diffusing nano-objects may be monitored while the surrounding conditions are modified (e.g., pH value or salinity). In such cases, it is essential to probe the same ensemble during the external change and not to exchange the liquid. This is hardly feasible with capillary-type structures, where the modifying substance must diffuse into the core through the ends to reach a specific concentration, often resulting in impractically long measurement times. In contrast, the sHCW enables direct and rapid sidewise access to the core. This advantage has previously been demonstrated by our group in locally structured light cages in the context of NTA, where solvent-induced changes in the hydrodynamic diameter distribution of a polymer-brush-enhanced nanoparticle system were dynamically monitored [link].

C. Pumping requirements and sample volume: Pressure-driven approaches typically require external pumping systems or at least syringes and thus large liquid reservoirs, which can be disadvantageous when only limited sample volumes are available. In contrast, the scaffold

waveguide enables passive, sidewise diffusion into the core without external pressure, making it particularly suitable for small sample volumes or sensitive analytes (c.f. our response to the eight comments of the Reviewer (R1.8)). Our approach also does not require additional components such as pumps, valves, or sealing interfaces, thereby reducing system complexity and potential sources of error or leakage.

D. Spatial homogeneity of analyte distribution: In capillary systems, pressure-driven filling can lead to concentration gradients across the core or along the waveguide. In contrast, sidewise access enables more uniform and rapid equilibration throughout the light/matter interaction region.

E. Compatibility with fragile or sensitive analytes: Pressure-driven filling can introduce shear forces that may alter or damage sensitive systems such as biological samples, soft matter, or nanoparticle assemblies (e.g. core-shell systems). The passive diffusion enabled by the scaffold waveguide avoids such perturbations, preserving the native state of the analyte.

F. Purpose of the presented diffusion experiments: The experiments presented in this study are intended to highlight the key advantage of the open cladding architecture for diffusion-dominated processes using a direct experimental approach. Beyond the aforementioned NTA scenario, sidewise access to the core is particularly relevant for gas-based applications at low pressures, where pressure-driven filling is not applicable and diffusion into the core becomes the limiting factor. In this scenario, the scaffold waveguide concept is highly promising, for example for alkali vapor-based quantum optics, which operate at low pressures and rely on efficient diffusion into the interaction region, as discussed in Sec. 1 of the Supplementary Information.

To account for the Reviewer comment, we have changed the text of the manuscript as follows: … The Photonic Scaffold approach offers distinct advantages for optofluidic and analytical applications, where efficient light–matter interaction and rapid analyte exchange are essential. Unlike tubular-type HCWs or ARROWs that suffer from limited core accessibility and slow analyte transport, the sHCW design enables unhindered lateral diffusion into the core, allowing rapid equilibration with the envi-ronment—crucial for time-sensitive and dynamic sensing. Simultaneously, it ensures strong light confinement in an exceptionally small interaction volume **in the order of nanoliters. It should be noted that in the current experimental implementation the total sample volume is not minimized, as the open waveguides are immersed in a macroscopic liquid reservoir. Nevertheless, the platform is inherently compatible with microfluidic integration (e.g. fiber interfacing on V-groove enhanced Si-chips [link]), where enclosing the Photonics Scaffold within a microfluidic chamber would enable a substantial reduction of the required sample volume to the microliter or even sub-microliter regime**. **Note that capillary systems, commonly used in liquid spectroscopy, typically require pressure-driven liquid exchange. In contrast, the openness of the Photonic Scaffold avoids using pressure, preventing mechanical stress and potential structural deformation, while enabling sidewise diffusion of relevant species into the core under controlled conditions. The sHCW further promotes more homogeneous and fast equilibration of the analyte distribution, reduces system complexity by eliminating the need for pumps or fluidic interfaces, and is particularly well suited for applications involving limited sample volumes or sensitive analytes.** In biomedicine, the platform can

detect specific molecular species [48] and dynamically monitor nanoscale processes via nanoparticle tracking [22]. Functional surface coatings [49], including bioreceptors and hydrophobic layers, provide capabilities such as selec-tive binding and clogging prevention. In the field of chemical sensing, the platform can detect industrial compounds, such as alcohols, ketones, and organic acids [50], support the monitoring of solvent purity in pharmaceutical [51] and semiconductor processes [52]; and enable the real-time tracking of reactions in microfluidic systems for kinetic and intermediate analysis [53]. …

R1.2: How robust is the scaffold structure against transverse fluid flow? Have the authors tested the maximum transverse flow velocity it can withstand? The authors also state that the scaffold waveguides can withstand cryogenic temperatures, but no supporting data are provided. Have they tried cooling down the structures, and do they survive the thermal stress involved?

We thank the Reviewer for this thoughtful comment and for raising the important aspect of transverse flow and a low temperature environment. In the following, the two points mentioned by the Reviewer are addressed separately.

A. Impact of transverse flow: This is an interesting aspect, and we agree that there will be a limit to the transverse flow that can be applied without affecting the structure. However, a systematic investigation of the flow regime at which the waveguide properties change or the structure detaches from the substrate is beyond the scope of the present study, as it would require extensive sample fabrication and a statistically meaningful analysis over a large number of samples. In addition, the relevant behavior is expected to strongly depend on fabrication parameters, since different writing strategies can result in varying mechanical stiffness, and surface treatments such as silanization or plasma processing can significantly influence adhesion to the substrate. A comprehensive study would also require coupled fluidic and mechanical simulations that take into account the geometry and material properties, as well as the definition of appropriate figure-of-merit quantities to enable generalizable conclusions. Altogether, this indicates that a rigorous investigation of transverse flow effects constitutes a dedicated study on its own and is therefore outside the scope of the present work. Nevertheless, the practical robustness of the Photonic Scaffold has been qualitatively demonstrated during our experiments. For instance, the structures remained fully intact and functionally stable during routine immersion and handling in liquid environments as described below. Notably, the waveguides survived the mechanical turbulence and fluidic shocks induced by the boiling of liquid nitrogen during the cryogenic tests, suggesting that the scaffold architecture possesses sufficient mechanical strength to withstand moderate fluidic disturbances.

B. Impact of cryogenic temperatures: We thank the Reviewer for raising this important point. In contrast to the transverse flow discussed above, the influence of cryogenic temperatures on the properties of the sHCW can be experimentally assessed straightforwardly by exposing the structures to a cryogenic environment, subsequently returning them to ambient conditions, and comparing their transmission characteristics before and after exposure. For this purpose, liquid nitrogen was used as a representative cryogenic medium. It is noted that, in the scientific community, cryogenic conditions generally refer to temperatures below -150°C (123 K), and liquid nitrogen falls well within this range, with a boiling point of approximately -196°C (77 K).

To assess the impact of exposure to liquid nitrogen on the fabricated sHCWs, two types of waveguides with different cladding-openness fractions (f = 0.20 and f = 0.68) were prepared and their spectral transmission was measured using the setup described in the manuscript. Subsequently, the samples were exposed to liquid nitrogen for a defined duration of 5 min, after which the optical characterization was repeated and compared to the initial measurements.
The results (Fig. RL1) show that the transmission spectra recorded after exposure to liquid nitrogen (dashed curves) closely match those obtained prior to exposure (solid curves), indicating that the low-temperature treatment only marginally affects the fabricated structures. For the case of high openness (Fig. RL1(b), f = 0.68), slight variations in transmission amplitude are observed, while the resonance positions remain unchanged, confirming that the structural integrity of the waveguide is preserved. Overall, these findings suggest that exposure to cryogenic environments does not induce optically relevant structural changes to the Photonic Scaffold, thereby supporting its suitability for low-temperature applications.

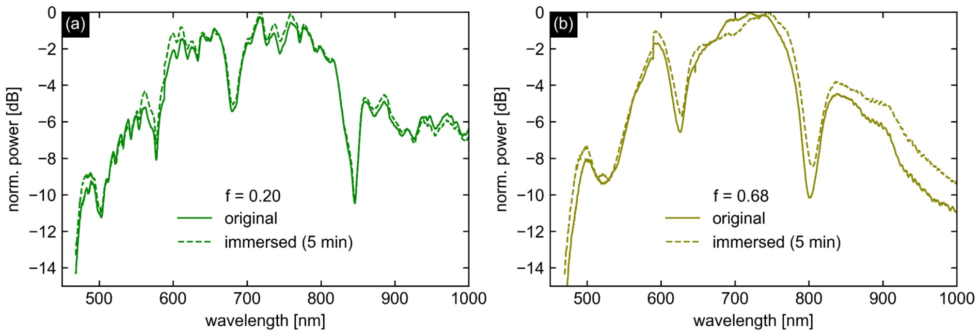

Fig. RL1: Measured spectral transmission of the fundamental core mode for freshly 3D nanoprinted sHCWs (type I, solid curves) compared to the same structures after immersion in liquid nitrogen for 5 min (dashed curves). Results are shown for two types of structures with different cladding-openness fractions: (a) f = 0.20 and (b) f = 0.68.

We thank the Reviewer for this valuable suggestion, which motivated this additional investigation, which will be addressed in detail in a future publication.

To address the Reviewer's comment, the corresponding discussion and figure have been included in the Supplementary Information, and the manuscript has been revised as follows:
… Note that the sample remained fully functional for over five years without signs of degradation [REF], highlighting the effectiveness of the nanofilm in preventing corrosion [REF]. The HCW architecture is also well suited for cryogenic environments, **which is supported by experimental verification provided in the Supplementary Information (Sec. 9), where the spectral transmission of selected sHCWs was measured before and after exposure to liquid nitrogen. The results show only minor amplitude variations while preserving the spectral positions of the resonances, confirming the structural integrity of the Photonic Scaffold under low-temperature conditions.** …

R1.3: Table 3: The comparison of the mode volume of the scaffold waveguide and the “Bulk”/diverging beam is quite biased by the beam waist parameter chosen for the free-space beam. The authors choose an input beam waist of 0.75 um, which results in a mode volume of 3671 nL (compared to 1.41 nL for the scaffold waveguide with a beam waist

of 9.47 um). In their table, the authors should also include a row showing the effective volume of a free-space beam with the same waist diameter as the scaffold waveguide (9.47 um). A quick check of Equation 7 suggests that this would result in an effective volume for the free-space beam that is two orders of magnitude smaller than for the tightly-focused beam currently shown in table 3. Note that the value for the less-diverging beam would still be 30-40 times larger than that of the scaffold waveguide, but it would, in my opinion, be a fairer way to compare its performance to that in a bulk experiment.

We thank the Reviewer for the comment regarding the comparison of the effective liquid volumes and the susceptibility of the bulk configuration to the assumed beam waist. While we agree that extending the comparison to a Gaussian beam with an input waist identical to that of the mode inside the sHCW is useful, we first clarify the rationale behind the chosen beam waist in the bulk configuration and then discuss the additional row included in the table.

A. Choice of beam waist for the bulk configuration: As shown in Sec. 6 of the Supplementary Information (c.f. Fig. 5), the divergence angle θ of the beam inside the water-filled chamber was experimentally determined by launching light into the chamber without a waveguide at the peak absorption wavelength (λ_p = 527 nm) and visualizing the beam profile via side-emitted fluorescence. The resulting intensity distribution along the x-direction was normalized to its maximum at each z-position, and values below exp(-2) ≈ 0.135 were cropped. The divergence angle θ is defined as the angle between the beam center axis and the boundary between cropped and uncropped regions, yielding θ = 5.86°. Assuming Gaussian beam propagation in water, this divergence corresponds to a beam waist of w_0 = 0.75μm, as stated in Tab. 3 of the main text. This justification of the beam waist was not included in the original manuscript, which may have suggested an arbitrary choice of divergence angle. The text has therefore been extended accordingly to clarify this point as shown below.

B. Additional configuration: As suggested by the Reviewer, we additionally consider a freely diffracting Gaussian beam at the input of the sHCW with a beam waist identical to that of the guided mode (w_0 = 9.47μm). In this case, the resulting output beam waist is 66μm, corresponding to an effective liquid volume of 24.4 nL, which is 17.3 times larger than that of the sHCW (1.4nL). These results have been included in Tab. 3 of the manuscript which now reads as follows (Tab. RL1):

Tab. RL1: Key parameters relevant for the diffusion experiments, including channel geometries, optical parameters, and diffusion times for three configurations presented in Fig. 8, along with an additional Gaussian beam configuration assuming a beam waist identical to that of the sHCW.

Configuration	t_e [min]	Channel diameter [μm]	Beam div. angle [°]	Input beam waist [μm]	Output beam waist [μm]	V_{eff} [nL]
HCW	0.57	26	0	9.47	9.47	1.41
Capillary	132.18	200	0	72.85	72.85	83.37

Bulk (no wg)	0.4	-	5.86	0.75	837.42	3671.86
Gaussian beam	-	-	0.76	9.47	66.99	24.4

To address the Reviewer's comment, the expanded table (Tab. RL1) and the corresponding text in the manuscript have been included in the manuscript as follows: … In the case of a guided mode, $z_R \rightarrow \infty$, leading to $V_{eff} = \pi \ w_0^2$ L. **In the following, the effective liquid volume of the sHCW (approximated as a cylindrical waveguide) is compared to that of the capillary (Tab. 3), demonstrating that light is confined to a remarkably small volume of approximately 1.4 nL, which is about 60 times smaller than in the capillary (83 nL). The situation of diffraction of an unconfined beam was also considered by measuring the divergence angle of the excitation beam in the absence of the sHCW (beam divergence angle is θ = 5.86 degrees, c.f. Sec. 6 of the Supplementary Information), yielding V_{eff} = 3671 nL, which is 2600 times larger than that of the sHCW. In addition, a freely diffracting Gaussian beam at the input of the sHCW with a beam waist identical to that of the guided mode (w_0 = 9.47 µm) was also considered. In this case, the resulting output beam waist is 66 µm, corresponding to an effective liquid volume of 24.4 nL, which is 17.3 times larger than that of the sHCW (1.4 nL).** These findings underline the unique capability of the sHCW to combine nearly unrestricted analyte diffusion with extraordinary light confinement - an exceptional advantage in diffusion-based sensing. …

…

Tab. 3: Key parameters relevant for the diffusion experiments, including channel geometries, optical parameters, and diffusion times for three configurations presented in Fig. 8**, along with an additional Gaussian beam configuration assuming a beam waist identical to that of the sHCW**.

We have also slightly extended Sec. 6 of the Supplementary Information to include the mathematical framework used to determine the divergence angle: … To experimentally determine this parameter, a trial chamber containing only the dye solution without a waveguide was prepared. By launching light at the peak absorption wavelength (λ_p = 527nm) into the chamber, the beam shape became visible via side-emitted fluorescence. To extract θ, a top-view image was recorded, and the intensity distribution along the *x*-direction was normalized to its respective maximum at each *z*-position. **At this stage, it is important to note that, since the camera integrates the emitted light along the line of sight, the measured intensity distribution *I*(*x*, *z*) represents the projection of the three-dimensional Gaussian beam profile, with the propagation direction *z*, the transverse coordinate *x*, and integration along the imaging axis *y*.**

$$I(x,z) = \int_{-\infty}^{+\infty} I(x,y,z)\, dy \propto \frac{1}{w(z)} \exp\left(-\frac{2x^2}{w(z)^2}\right)$$

As this integration over the y-axis modifies only the prefactor, the exponential term governing the transverse profile remains unchanged, allowing the beam width to be determined from the well-known intensity reduction level exp(-2) ≈ 0.135. Therefore, intensity values below 0.135 were cropped and visualized in a different color (Fig. 5). The divergence angle θ is then defined as the angle between the beam center axis (magenta dashed-dotted line) and the boundary between cropped and uncropped regions (magenta

dashed line), yielding θ = 5.86°. Note that …

R1.4: What are the losses in liquid-immersed waveguides? Were these measured/estimated, and are they larger/smaller than the air-guided waveguides?

We thank the Reviewer for raising this important point regarding the losses in liquid-immersed waveguides. We agree that a direct comparison between air-guided and liquid-immersed conditions is essential for assessing the applicability of the concept with analytics and optofluidics.
To address this comment from an experimental perspective, additional measurements were performed on a sHCW within a liquid environment. Specifically, the sample (f = 0.68, d_c = 26 µm) was immersed in water, and the losses were determined using the same methodology as for the measurements in air. The results (including the new core-size dependence measurements performed in response to the seventh comment of the Reviewer (R1.7)) have been incorporated into Tab. 1 of the manuscript (c.f. Tab. RL2).
The measurements show that, in a water environment, the sHCW show loss values between 0.15 and 0.39 dB/mm at visible wavelengths, demonstrating that low-loss guidance is preserved under liquid conditions. For the longest wavelength considered, increased loss is observed due to the onset of strong NIR absorption of water. Note that, due to the modified spectral response of the sHCW, resulting from the change in refractive index contrast, different wavelengths are selected for the water case.

Tab. RL2: Overview of experimentally determined modal losses for Type I Photonic Scaffolds with two core extents at ultrahigh cladding-openness fraction (f = 0.68, (a) d_c = 22 µm, (b) d_c = 26 µm) compared to a structure with moderate openness (f = 0.20, d_c = 26 µm) for air and water environment. The loss values are evaluated at selected wavelengths (given in the column labeled λ_s) within transmission bands of the sHCW considered; for the waveguide in air with f = 0.68, the spectral positions are indicated by vertical blue dotted lines in Fig. 6.

f	environment	d_c [µm]	λ_s [nm]	γ [dB/mm]
0.68	air	22	465, 530, 652, 807	0.59, 0.66, 0.86, 0.93
0.68	air	26	465, 530, 652, 807	0.42, 0.56, 0.53, 0.72
0.68	water	26	460, 678, 990	0.15, 0.39, 0.95
0.2	air	26	496, 572, 637, 740	0.27, 0.40, 0.42, 0.46

In response to the Reviewer's comment, we have revised the manuscript by substituting Tab. 1 with Tab. RL2 and implementing the following changes to the main text.

… Note that these values are on the same order or even lower than those of comparable on-chip HCWs with closed cladding geometries [REF]. **As discussed in Sec. 8 of the Supplementary Information, transmission-related benchmark quantities have been determined by measuring the optical power at different locations within the experimental configuration. The resulting coupling efficiencies at two selected wavelengths within the corresponding transmission bands are on the order of ≈23–25%. These values can be further improved in future experiments, for example by**

fiber interfacing in a V-groove type chip environment [link] or by directly printing sHCWs on fibers [link], optimizing mode matching via tailored beam-shaping optics, or by integrating on-chip micro-optics or computationally optimized couplers for enhanced coupling performance. In addition, measurements performed in a water environment for an sHCW with f = 0.68 and d_c = 26µm yield loss values between 0.15 dB/mm and 0.39 dB/mm at visible wavelengths, demonstrating that low-loss guidance is also preserved under liquid conditions. For the longest wavelength considered, increased loss is observed due to the onset of strong NIR absorption of water. Note that, due to the modified spectral response of the sHCW that results from the change in refractive index contrast, different wavelengths are selected for the evaluation of the water case. …

… **As demonstrated by the water-based measurements (Tab. 1),** the Photonic Scaffold approach offers distinct advantages for optofluidic and analytical applications, where efficient light–matter interaction and rapid analyte exchange are essential. …

R1.5: In the loss spectra in Fig 7, why is there an absorbance offset in the bulk absorption spectra in the long-wavelength region? Was this offset reproducible, or is it due to a baseline drift?

We thank the Reviewer for the comment regarding the offset observed in the bulk absorption data of the chamber in absence of the waveguide. To clarify this point, additional measurements were performed using a reference cuvette, and the raw transmission data of the sHCW, the chamber without the waveguide, and the cuvette are compared in Fig. RL2.

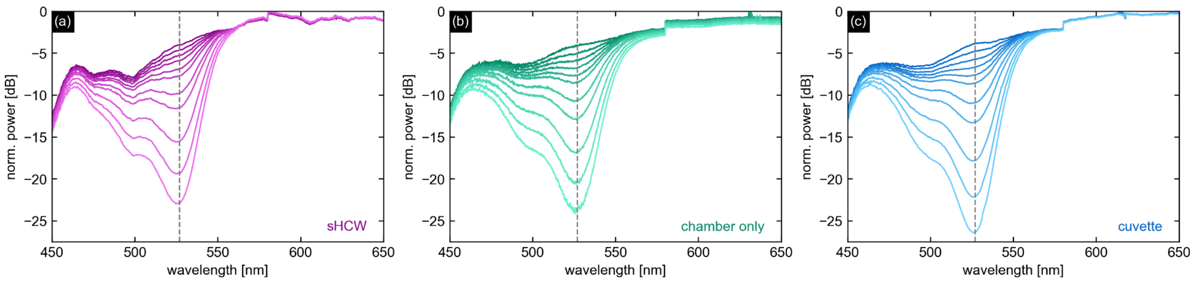

Fig. RL2: Normalized transmission spectra in the visible range for varying Rhodamine 6G (R6G) concentrations. (a) sHCW (type I, f = 0.68, waveguide length: 9.8mm, chamber length: 10mm), (b) chamber-only configuration (chamber length: 10mm), and (c) cuvette reference (length: 10mm). Concentrations range from 0 µM (darkest) to 25 µM (brightest): 0, 1, 2, 3, 4, 5, 7.5, 10, 15, 20, and 25 µM. The dashed vertical lines indicate the main absorption peak of R6G (λ_0 = 527nm).

It should be noted that, for each configuration, the transmission data were normalized to the respective maximum of the measurement set. Inspection of the raw data clearly shows that the offset occurs exclusively in the chamber-only configuration, while it is absent in both the cuvette measurements and in the waveguide-based investigations. This confirms that the effect originates from the chamber itself rather than from the measurement procedure. Additional independent measurements of the empty chamber (not shown) reproduced the same behavior, further supporting this conclusion. Although the exact origin of this effect remains unclear, it is important to emphasize that it does not appear in the measurements involving the waveguides and is therefore not relevant to the main conclusions of this work.

To address the Reviewer's comment, this point has been clarified in the revised manuscript as follows: … The resulting spectra match well (Fig.7(c)), confirming the applicability of the sHCW for optofluidic-based integrated absorption spectroscopy. **It should be noted that, as confirmed by additional cuvette-based reference measurements, the offset of the molar attenuation coefficient for the reference measurements without waveguide (green curve in Fig. 7(c)) outside the dye absorption region originates from the chamber itself rather than from the measurement procedure.** …

R1.6: On page 12 (Figure 8) the authors claim that the temporal behaviour of the scaffold waveguide is "nearly identical" to that of the bulk experiment. Considering the logarithmic time axis, however, there is still a 1.5 times difference in the time it takes for the absorbance to reach 50% of its peak value, which I would not describe as nearly identical. Please rephrase.

We thank the Reviewer for this important comment and for pointing out the potential overstatement. We agree that the term "nearly identical" may be misleading when considering the logarithmic time axis and the observed difference in characteristic diffusion times.
To address this, we have revised the text to more accurately reflect the experimental findings. While the absolute difference between the characteristic times (sHCW: t_e = 0.57 min, bulk: t_e = 0.40 min) corresponds to a factor of approximately 1.5, both values remain within the same sub-minute timescale, indicating rapid diffusion in both cases.

To account for the Reviewer's comment, we have changed the text accordingly: … The results indicate fast diffusion (< 1min) **for both configurations, with comparable characteristic times for the sHCW and waveguide-free measurements (sHCW: t_e = 0.57 min, bulk: t_e = 0.40 min, Tab. 3), indicating that diffusion in the scaffold is only slightly slower compared to the bulk behavior.** …

R1.7: On page 14, the authors write: "By comparison, conventional membrane-HCWs with smaller cores without openings show higher losses (3.2–3.9 dB/mm and ~10 dB/mm), highlighting the efficiency of our open-membrane design". Because the membrane - HCWs features smaller cores, which always result in higher waveguide losses, this result does, in my opinion, not highlight the "efficiency of the open-membrane design". To support this statement, losses of open and closed membrane waveguides with the same core diameter should be compared. Please include such data or rephrase the statement. Please include the core diameter of the (closed) membrane waveguide in the manuscript.

We thank the Reviewer for the comment regarding the loss comparison of structures with similar geometry. We agree that a quantitative comparison should be made between structures with identical core dimensions but different openness fractions, and we acknowledge that the corresponding statement in the manuscript was not fully accurate.

To address this point, additional experiments were performed, including the fabrication and characterization of a new set of sHCWs with a significantly reduced openness fraction (f = 0.20) and a core extent of d_c = 26μm (Fig. RL3).

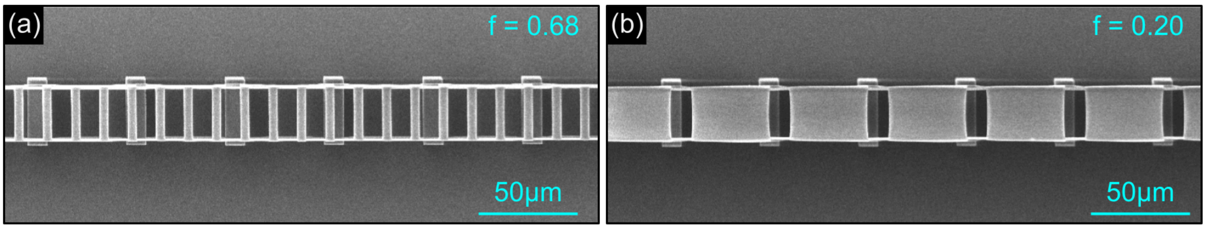

Fig. RL3: Scanning electron microscope (SEM) images of fabricated Type I sHCWs with different cladding-openness fractions used for the loss measurements. (a) Structure with ultrahigh openness (*f* = 0.68), and (b) structure with reduced openness (*f* = 0.20). Both waveguides exhibit comparable core dimensions (d_c ≈ 26 µm) and are used to investigate the impact of openness fraction and liquid immersion on modal loss. Scale bars: 50 µm.

The results (including the water-based measurements performed in response to the fourth comment of the Reviewer (R1.4)) have been incorporated into Tab. 1 of the manuscript (c.f. Tab. RL2), showing slightly lower loss values for the structure with reduced openness, while all investigated structures maintain losses below 1 dB/mm within the transmission bands, in agreement with the simulations presented in the manuscript.

To account for the Reviewer's comment, the manuscript has been revised by replacing Tab. 1 with Tab. RL2, including Fig. RL3 into the Supplementary Information and by incorporating the corresponding changes in the text as follows:

… The resulting losses at the selected wavelengths (vertical blue dotted lines in Fig.6) are presented in Tab. 1, with values ranging from 0.5 dB/mm to 0.9 dB/mm for both core extensions considered. **For comparison, an additional set of sHCWs with a reduced openness fraction (*f* = 0.20) and a core extent of d_c = 26µm was fabricated and optically characterized (see Sec. 7 of the Supplementary Information), revealing slightly lower loss values for the reduced-openness structure, while all investigated configurations maintain losses below 1 dB/mm within the transmission bands in agreement with simulations presented before.** Note that …

… These values are among the lowest reported for anti-resonant on-chip HCWs, demonstrating the effectiveness of our design. By comparison, conventional membrane-HCWs with smaller cores without openings show higher losses (3.2–3.9 dB/mm and ~10 dB/mm), highlighting the efficiency of our open-membrane design. **Moreover, the comparison of measured loss values for geometries with different openness fractions (Tab. 1) clearly demonstrates that even ultrahigh openness does not introduce significantly increased attenuation, as losses remain below 1 dB/mm within the transmission bands, thereby confirming the feasibility of the concept of the Photonic Scaffold.** While the light cage concept provides side access, it requires a larger core area and more complex fabrication. Our sHCW design uniquely combines an ultrahigh cladding-openness fraction with extremely low loss and full side access to the core, making Photonic Scaffolds particularly promising for applications requiring both efficient light guidance and direct light-matter interaction with diffusive species. …

R1.8: On page 16, the authors write:" Simultaneously, it ensures strong light confinement in

an exceptionally small interaction volume, enabling nanoliter-scale, high-sensitivity detection with minimal sample consumption." While I agree that the interaction volume is very small, it is not correct to say that their current approach minimises sample consumption. In their liquid experiments, the open waveguides are immersed in 1 mL of sample, of which only a small fraction enters the waveguide core. If the authors were to minimise their sample volume, the open waveguide would need to be enclosed within a slightly larger microfluidic channel. The resulting structure would then look very similar to optofluidic hollow core fibres. Please comment on this in the manuscript.

We thank the Reviewer for this insightful comment and for highlighting the distinction between interaction volume and total sample consumption. We agree that, in the current experimental implementation, the scaffold waveguides are immersed in a comparatively large liquid reservoir (on the order of mL), and thus the total sample consumption is not minimized.

Our original statement was intended to emphasize the extremely small light–matter interaction volume (on the order of nL), which is a key advantage for sensitivity and efficient light–matter coupling. However, we acknowledge that this does not directly translate to minimal total sample usage in the present experimental configuration.

Importantly, the platform discussed in this study is inherently compatible with microfluidic integration, which would allow a substantial reduction of the required sample volume. As also suggested by the Reviewer, enclosing the open scaffold waveguide within a microfluidic channel represents a natural next step. In fact, we have already demonstrated related integration approaches in previous work, where light cages were interfaced with optical fibers in on-chip V-groove configurations enabling compact and aligned optofluidic systems (Fig. RL 4(a)) [link]. Building on this, current developments in our research group aim at directly integrating a 3D nanoprinted microfluidic chamber around the hollow-core waveguide in the same fabrication run (an idea of such a chamber that includes a light cage structure is shown in Fig. RL4(b)), which would reduce the required liquid volume to the microliter regime. For example, the liquid volume inside such a chamber was estimated to be on the order of 100 nL, assuming realistic parameters of such a cell (length 1 cm, height 50 µm, width 100 µm, V-groove depth 100 µm, V-groove width 100 µm).

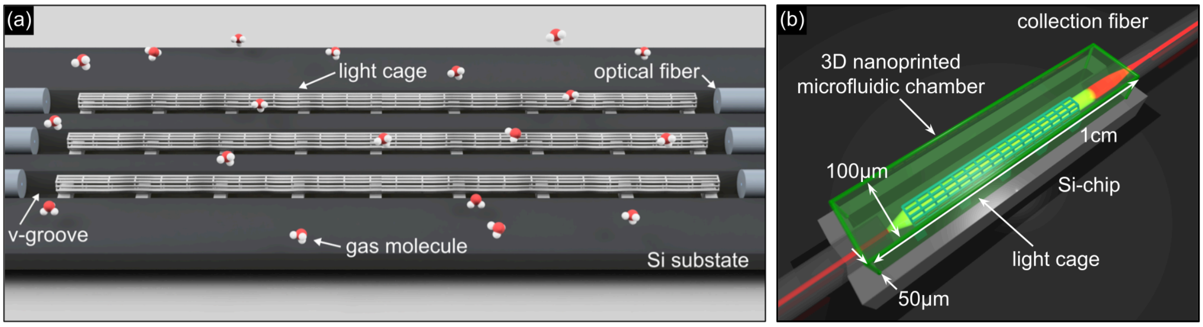

Fig. RL4: Conceptual idea for microfluidic integration of on-chip hollow-core waveguides. (a) Previously demonstrated on-chip configuration in which light cage structures are integrated with optical fibers using V-grooves on a silicon substrate, enabling compact and aligned optofluidic operation [link]. (b) Proposed implementation of a fully integrated 3D nanoprinted microfluidic chamber surrounding a light cage, fabricated in a single step on a silicon chip and interfaced with collection fibers;

representative chamber dimensions are indicated.

We note that, while such integration significantly reduces the sample volume within the sensing region, additional volumes in fluidic connections (e.g., tubing and reservoirs) remain a general consideration for all microfluidic systems.

To address the Reviewer’s comment, we have revised the manuscript as follows: … Simultaneously, it ensures strong light confinement in an exceptionally small interaction volume **in the order of nanoliters. It should be noted that in the current experimental implementation the total sample volume is not minimized, as the open waveguides are immersed in a macroscopic liquid reservoir. Nevertheless, the platform is inherently compatible with microfluidic integration (e.g. fiber interfacing on V-groove enhanced Si-chips [link]), where enclosing the Photonics Scaffold within a microfluidic chamber would enable a substantial reduction of the required sample volume to the microliter or even sub-microliter regime. Note that capillary systems, commonly used in liquid spectroscopy, typically require pressure-driven liquid exchange. In contrast, the openness of the Photonic Scaffold avoids using pressure, preventing mechanical stress and potential structural deformation, while enabling sidewise diffusion of relevant species into the core under controlled conditions. The sHCW further promotes more homogeneous and fast equilibration of the analyte distribution, reduces system complexity by eliminating the need for pumps or fluidic interfaces, and is particularly well suited for applications involving limited sample volumes or sensitive analytes.** In biomedicine, …

Response to comments of Reviewer #2

The authors present a novel geometry for an on-chip hollow-core waveguide. Hollow-core waveguides have been around for a while, and this group are leading the research. The geometry they present here is truly novel and I am impressed by the high performance it exhibits despite the very open structure. I agree that the open structure is very exciting, as it gives easy access to the analyte and changes within it; the diffusion work shown in fig. 8 clearly highlights this capability.

We appreciate the Reviewer's careful reading of the manuscript, the positive feedback, and the valuable comments. We have addressed all points in detail below, providing a point-by-point response and indicating the corresponding revisions made in the manuscript.

I only have some minor comments:

R2.1: I do not understand why they separate between type I and type II. Clearly, there are thousands of variants possible with this geometry. Reading the paper, I find the discussion of the two types distracting, and I don't really understand why the two types are needed. In table 4, I assume there is a typo, as the first two rows both refer to type I; should this be type I (first row) and type II (second row)? If so, the performance is very similar, so again, why do we need to see the difference? In conclusion: If you want to keep the two types, make the distinction very clear: "Type 1 has these unique properties, while type II offers a different trade-off leading to a different set of unique properties." As it stands, type 2 is only introduced to highlight the advantage of the symmetry of type I. If that's all, please move type

II to the SI in its entirety.

We thank the Reviewer for this thoughtful and constructive comment and for pointing out the need to better justify the inclusion and distinction of Type I and Type II geometries. We also thank the Reviewer for identifying the typo in Tab. 4, which has been corrected accordingly.

Regarding the distinction between Type I and Type II, we agree that a clearer motivation and differentiation is useful. The two geometries are not intended to represent an exhaustive classification, but rather to illustrate two representative design principles within the large parameter space enabled by the Photonic Scaffold concept. Specifically, Type I features a symmetric membrane arrangement that fully encloses the core within each period, whereas Type II introduces a half-period shift between vertical and horizontal openings, resulting in a different longitudinal modulation of the structure.

The inclusion of Type II serves several purposes:

(i) it demonstrates that structural variations beyond the symmetric Type I configuration can be realized while remaining structural integrity,
(ii) it highlights the capability of 3D nanoprinting to implement complex 3D architectures with longitudinal variations (alternating sideaccess) that are difficult to achieve with conventional planar fabrication techniques,
(iii) it shows that light guidance persists even in Photonic Scaffolds with higher structural complexity, albeit with slightly reduced performance compared to Type I, and
(iv) it provides a transmission window that matches the requirements of the single-photon transmission experiments.

We agree with the Reviewer that, in the original version, this distinction and motivation were not sufficiently clear. We have therefore revised the manuscript to explicitly state the conceptual difference between the two types and to clarify that Type I represents the optimized, symmetric configuration, while Type II mainly serves as a proof-of-concept for more complex structural variations enabled by 3D nanoprinting, illustrating robustness and design flexibility of this photonic platform:

… To ~~systematically~~ explore the impact of introducing openings into the membranes of the sHCW, two types of Photonic Scaffold geometries **with different longitudinal distributions of the openings** are defined and investigated experimentally: …

… Notably, the structure with a = 11µm and Λ = µm (blue curve) reveals an almost identical spectral transmission as the low cladding-openness fraction reference (a = 2µm and Λ = 100µm, orange curve), clearly demonstrating that proper waveguide design enables ultrahigh cladding-openness fraction sHCWs with transmission performance comparable to their closed counterparts **even for Photonic Scaffolds geometries that have a higher level of structural complexity.** …

… The third demonstration of the application relevance of the Photonic Scaffold approach assesses its applicability in photonic quantum technologies by measuring single-photon transmission. Here, emission from a single semiconductor quantum dot (QD) was coupled into a type II sHCW (d_c = 22µm, Λ = 19.5µm, f = 0.67) and analyzed. **Note that a Type II sHCW is chosen here because its transmission window matches the required**

wavelength, avoiding the need for additional sample design. The QDs, embedded in high-brightness micropillar cavities, …

… When comparing the two types of sHCWs addressed in this study, both exhibit roughly identical transmission levels. However, Type II sHCWs display less regular transmission patterns and a slight reduction in the near-infrared region, indicating that a symmetric membrane arrangement used for Type I sHCW geometry may be more advantageous for efficient light transportation. **Note that the investigation of Type II structures serves several objectives: to demonstrate mechanical integrity beyond the symmetric Type I design; to illustrate the capability of 3D nanoprinting to implement 3D architectures with longitudinal variations (axial alternation of openings); and to confirm light guidance in Photonic Scaffolds of higher structural complexity, i.e. with a longitudinally opening distribution that is challenging to realize with other fabrication approaches.** …

Given this broader conceptual role, we believe that retaining Type II within the main manuscript is sufficiently justified. From our perspective, the revised text now includes a clear motivation for the inclusion of the Type II geometry and addresses the Reviewer's concern regarding potential distraction. We hope that the Reviewer finds our argumentation reasonable and acceptable.

The Reviewer also raised a comment regarding the first entries in Tab. 4. Here we would like to mention that there is not a typo in the table, as the two rows refer to two different Type I sHCWs with different core sizes (top row: 22 µm x 22 µm, second row: 26 µm x 26 µm). Therefore, the entries in the table have been kept unchanged, and the table caption has been revised as follows: … Comparison of key performance indicators for the **two type I** sHCWs **characterized** in this work **(Fig. 6)** alongside state-of-the-art on-chip HCWs from the literature, including guiding mechanism, core geometry and area, optical losses, spectral operating range, and side access capability. …

R2.2: Can you please specify the coupling efficiency/insertion loss? I assume efficiency is high, but that would be good to confirm.

We thank the Reviewer for this important comment and for pointing out the relevance of specifying coupling efficiency and insertion loss. We agree that providing these values would be helpful to further assess the performance of the experimentally used configuration.
To address the Reviewer's comment and considering the different definitions of coupling efficiency and insertion loss used in the literature, we provide below (Fig. RL5) a detailed schematic indicating the relevant power levels at the different positions within the experimental setup, allowing to calculate the corresponding benchmark quantities in a consistent manner. This approach ensures maximum transparency regarding the various definitions of coupling efficiency and enables a clear benchmarking of the results.

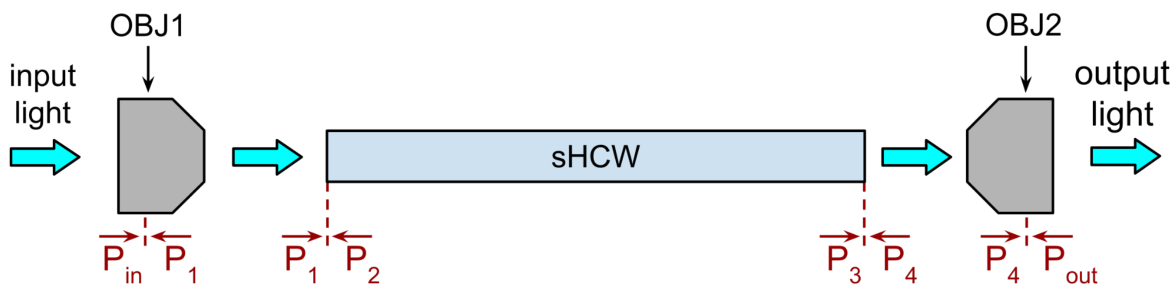

Fig. RL5: Schematic representation of the optical powers at different locations in the experimental configuration (light gray: incoupling lens, light blue: sHCW). The cyan arrows refer to the direction of the light (from left to right). Note that the direction of the red arrows indicates the side of the respective interface (e.g., the arrow for P_1 refers to the power on the left-handed side of the input of the sHCW).

In the following, the different efficiencies are determined at two selected wavelengths within the corresponding transmission band. Assuming that all light exiting the waveguide is collected by the output objective, the output power inside the sHCW P_3 is determined from the measured output power P_{out} and the characterized transmission of the objective. This approach assumes that the power at the end facet of the waveguide is identical to the power immediately outside the waveguide, i.e., that interface reflections are negligible ($\eta_{oi} = P_4/P_3 \approx 1$), which is reasonable given the negligible refractive index discontinuity at the waveguide output. The input power inside the waveguide P_2 is then calculated from P_3 using the measured propagation loss, which in the present case (sample length 5 mm) are approximately 0.4 dB/mm at λ_0 = 546 nm and 0.62 dB/mm at λ_0 = 712 nm. The following table (Tab. RL3) summarizes the resulting efficiencies, including total setup transmission, device transmission, coupling efficiency, and the corresponding insertion losses for both wavelengths.

Tab. RL3: Overview of efficiencies and loss metrics used to quantify the optical performance of the experimental configuration for a sample of length 5mm. Definitions are given in terms of the corresponding power levels at specific locations in the system based on the power definitions introduced in Fig. RL5, and values are evaluated at two selected wavelengths (λ_1 = 546nm , λ_2 = 712nm).

name of efficiency	symbol	definition	values @ λ_0 = 546nm	values @ λ_0 = 712nm	
total setup	η_s^{tot}	P_{out}/P_{in}	0.101	0.093	measured
input lens	η_{il}	P_1/P_{in}	0.823	0.867	measured
output lens	η_{ol}	P_{out}/P_4	0.835	0.879	measured
total device	η_d^{tot}	$P_4/P_1 = \eta_s^{tot}/\eta_{il}$	0.147	0.121	calculated
output interface	η_{oi}	P_4/P_3	≈ 1	≈ 1	assumed
coupling efficiency	η_c	P_2/P_1	0.234	0.248	calculated
insertion loss (setup)	IL_s	$-\ 10\log_{10}\left(\eta_s^{tot}\right)$	~ 10 dB	~ 10 dB	calculated
insertion loss (device)	IL_d	$-\ 10\log_{10}\left(\eta_d^{tot}\right)$	~ 8 dB	~9 dB	calculated

The total setup transmission is on the order of ~10%, corresponding to an insertion loss of

approximately 10 dB, which includes all contributions from coupling optics and the waveguide device. By isolating the device contribution, the total device transmission is found to be ≈15% (546 nm) and ≈12% (712 nm), corresponding to insertion losses of ≈8 dB and ≈9 dB, respectively. The resulting coupling efficiency into the waveguide using the current experimental configuration is about ≈23–25%. Approaches to improve this moderate coupling efficiency include fiber interfacing either in a V-groove type chip environment [link] or by directly printing sHCWs on fibers [link], optimizing mode matching via tailored beam-shaping optics (e.g., aspheric or diffractive elements), or integrating on-chip micro-optics or computationally optimized couplers for enhanced coupling performance.

To account for the Reviewer's comment, Fig. RL5 and Tab. RL3 including the relevant text have been included in the Supplementary Information, and the text of the main manuscript has been extended accordingly as follows: … Note that these values are on the same order or even lower than those of comparable on-chip HCWs with closed cladding geometries [REF]. **As discussed in Sec. 8 of the Supplementary Information, transmission-related benchmark quantities have been determined by measuring the optical power at different locations within the experimental configuration. The resulting coupling efficiencies at two selected wavelengths within the corresponding transmission bands are on the order of ≈23–25%. These values can be further improved in future experiments, for example by fiber interfacing in a V-groove type chip environment [link] or by directly printing sHCWs on fibers [link], optimizing mode matching via tailored beam-shaping optics, or by integrating on-chip micro-optics or computationally optimized couplers for enhanced coupling performance. In addition, measurements performed in a water environment for an sHCW with f = 0.68 and d_c = 26µm yield loss values between 0.15 dB/mm and 0.39 dB/mm at visible wavelengths, demonstrating that low-loss guidance is also preserved under liquid conditions. For the longest wavelength considered, increased loss is observed due to the onset of strong NIR absorption of water. Note that, due to the modified spectral response of the sHCW that results from the change in refractive index contrast, different wavelengths are selected for the evaluation of the water case.** …

Overall, a well-written paper. I appreciate the clarity of description without the overuse of superlatives one often sees these days.

We thank the Reviewer for the kind comment and fully agree that the overuse of superlatives is generally not appropriate for presenting scientific results.
